# Twenty years of evolution and diversification of digitaria streak virus in Digitaria setigera

**DOI:** 10.1093/ve/veab083

**Published:** 2021-10-13

**Authors:** Sergio Ortega-del Campo, Ioana Grigoras, Tatiana Timchenko, Bruno Gronenborn, Ana Grande-Pérez

**Affiliations:** CNRS, Institut des Sciences du Végétal, Gif-sur-Yvette 91198, France; Instituto de Hortofruticultura Subtropical y Mediterránea ‘La Mayora’ (IHSM-UMA-CSIC), Área de Genética, Facultad de Ciencias, Campus de Teatinos, Málaga 29071, Spain; CNRS, Institut des Sciences du Végétal, Gif-sur-Yvette 91198, France; CEA, CNRS, Institute for Integrative Biology of the Cell (I2BC), Université Paris-Saclay, Gif-sur-Yvette 91198, France; CNRS, Institut des Sciences du Végétal, Gif-sur-Yvette 91198, France; CEA, CNRS, Institute for Integrative Biology of the Cell (I2BC), Université Paris-Saclay, Gif-sur-Yvette 91198, France; Instituto de Hortofruticultura Subtropical y Mediterránea ‘La Mayora’ (IHSM-UMA-CSIC), Área de Genética, Facultad de Ciencias, Campus de Teatinos, Málaga 29071, Spain

**Keywords:** geminiviruses, mastreviruses, digitaria streak virus, viral quasispecies, evolutionary rate, substitution rate, mutation frequency, substitution bias, genetic variability

## Abstract

Within the family Geminiviridae, the emergence of new species results from their high mutation and recombination rates. In this study, we report the variability and evolution of digitaria streak virus (DSV), a mastrevirus isolated in 1986 from the grass *Digitaria setigera* in an island of the Vanuatu archipelago. Viral DNA of DSV samples was amplified from *D. setigera* specimens, derived from the naturally infected original plant, which were propagated in different laboratories in France and Italy for more than 20 years. From the consensus sequences, the nucleotide substitution rate was estimated for the period between a sample and the original sequence published in 1987, as well as for the period between samples. In addition, the intra-host genetic complexity and diversity of 8 DSV populations with a total of 165 sequenced haplotypes was characterized. The evolutionary rate of DSV was estimated to be between 1.13 × 10^−4^ and 9.87 × 10^−4^ substitutions/site/year, within the ranges observed in other single-stranded DNA viruses and RNA viruses. Bioinformatic analyses revealed high variability and heterogeneity in DSV populations, which confirmed that mutant spectra are continuously generated and are organized as quasispecies. The analysis of polymorphisms revealed nucleotide substitution biases in viral genomes towards deamination and oxidation of single-stranded DNA. The differences in variability in each of the genomic regions reflected a dynamic and modular evolution in the mutant spectra that was not reflected in the consensus sequences. Strikingly, the most variable region of the DSV genome, encoding the movement protein, showed rapid fixation of the mutations in the consensus sequence and a concomitant dN/dS ratio of 6.130, which suggests strong positive selection in this region. Phylogenetic analyses revealed a possible divergence in three genetic lineages from the original Vanuatu DSV isolate.

## 1. Introduction

The family Geminiviridae comprises one of the largest and most diverse groups of plant viruses. Among the geminiviruses, there are important plant pathogens that cause diseases in economically important plants in most tropical and subtropical regions of the world (Zerbini et al., 2017; Lefeuvre et al., 2019).

Geminiviruses are single-stranded DNA (ssDNA) viruses and display high mutation and recombination rates, which allow their populations to experience high variability and genetic heterogeneity (Duffy and Holmes 2008; Wu et al., 2015; Mabvakure et al., 2016; Sánchez-Campos et al., 2018; Juárez et al., 2019; Pinto et al., 2021). These features, similar to those of RNA viruses (Domingo, Sheldon, and Perales 2012; Elena, Fraile, and García-Arenal 2014; Lefeuvre et al., 2019), endow ssDNA viruses with great adaptive capacity. The similarities in variability and evolutionary rate between ssDNA and RNA viruses occur despite the differences of the polymerases used for their replication. ssDNA viruses use cellular DNA polymerases to replicate in the nucleus through a rolling circle (RCA) mechanism (Hanley-Bowdoin et al., 2013; Wu et al., 2020). In contrast, RNA viruses replicate their genomes by RNA-dependent polymerases, expressed from their own genomes and with low fidelity due to the lack of error correction mechanisms (Jenkins et al., 2002; Hicks and Duffy 2014). The genetic variability that characterizes ssDNA and RNA viruses is a consequence of their high spontaneous mutation rates and allows them to organize themselves into populations compatible with the concept of quasispecies (Isnard et al., 1998; Ge et al., 2007; Grigoras et al., 2010; Sánchez-Campos et al., 2018; Juárez et al., 2019), similar to those existing in RNA viruses (Domingo, Sheldon, and Perales 2012). Viral quasispecies are closely related genomes subjected to mutations, competition, and selection and allow viruses to adapt to different environments and hosts warranting their replicative success and survival (Domingo et al., 2006; Domingo 2016).

Digitaria streak virus (DSV) is a virus species of the genus *Mastrevirus*, one of the many genera of classified geminiviruses (Zerbini et al., 2017; Zhao et al., 2019; Koonin et al., 2020). DSV is serologically related to maize streak virus (MSV) (Dollet et al., 1986; Accotto et al., 1993), and its natural host is *Digitaria setigera*, a perennial grass species (East Indian crabgrass) native to Vanuatu, in the South Pacific. Donson et al. sequenced the DSV genome as one of the first geminiviruses and assembled a consensus sequence (Donson et al., 1987). At that time, it was not easily possible to take into account numerous sequence variants, and the concept of quasispecies was just being developed and applied to RNA viruses. The DSV genome consists of a circular DNA molecule that contains 2,701 nucleotides and four genes organized in two bidirectional transcription units (Fondong 2013): in the viral sense, the *CP* gene (*V1*), which encodes the capsid protein, and the *MP* gene (*VP*), which encodes the movement protein; in the complementary sense, the proteins required for replication Rep (*C1–C2*) and RepA (*RepA* gene or *C1*) are encoded. Rep is expressed by open reading frames *C1* and *C2* via a spliced messenger RNA. The transcription units are separated by two non-coding sequences: a short intergenic region (SIR) and a long intergenic region (LIR). Both regions are involved in the initiation of replication and transcription of the genome (Fondong 2013; Zerbini et al., 2017).

DSV and its host *D. setigera* represent a unique virus–host pathosystem since infected plants can be easily propagated vegetatively and this way maintained for decades or longer without losing viability of the virus, a much longer period than annual or short-lived perennial hosts of most plant viruses. Taking advantage of this experimental virus–host system, in this study, we examined the patterns that determine the organization, genetic variability, and evolution of DSV. We cultivated and propagated DSV-infected *D. setigera* plants for more than 20 years and obtained fifteen independent samples. Genome sequences of the respective viral populations were obtained and analysed. Mutant spectra were characterized, and phylogenetic analyses were performed. Our results show that the DSV population is complex, diverse, and rapidly evolving, consistent with the concept of genetic quasispecies.

## 2. Materials and methods

### 2.1 Plants and virus

*Digitaria setigera* specimens were derived from an infected plant collected at Saraoutou, Espirito Santo island, Vanuatu, from which DSV was isolated and sequenced for the first time (Dollet et al., 1986; Donson et al., 1987).

**Figure 1.**
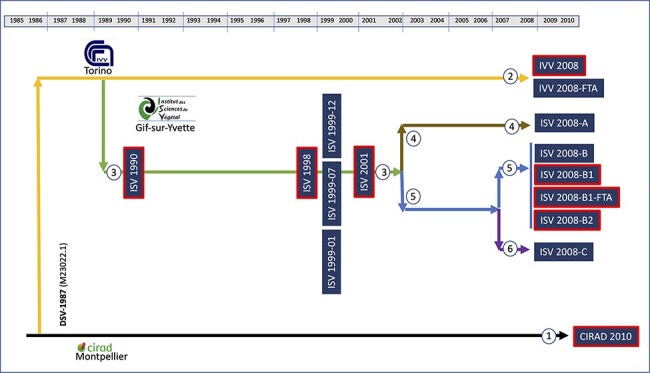
Schematic representation summarizing the DSV evolution in *D. setigera* over more than 20 years as a ‘sample tree’. The original plant was collected from Vanuatu and used to derive the first consensus sequence published by Donson et al. (1987). A part of this plant was taken to CIRAD (Montpellier, France) and from there to IVV (Torino, Italy). A specimen of DSV-infected *D. setigera* was taken to the ISV (Gif-sur-Yvette, France), where samples from different plants were obtained through vegetative propagation. Coloured arrows and circles with numbers show different plant lineages. Boxes with red borders represent samples for which consensus sequences were obtained by aligning the sequences of molecular clones (mutant spectra). For other samples, consensus sequences were obtained by aligning the sequences of RC-amplified ligation products.

A part of this original DSV-infected plant was taken to the Centre de Coopération Internationale en Recherche Agronomique pour le Développement (CIRAD, Montpellier, France) and from there to the Istituto di Virologia Vegetale (IVV, Torino, Italy). After 3 years of virus propagation in Italy, a specimen of *D. setigera* was taken by G. P. Accotto from the IVV to the Institut des Sciences du Végétal (ISV, now I2BC) of CNRS in Gif-sur-Yvette, France. Plants were maintained by vegetative propagation for more than 20 years in these three centres (Fig. 1). All *D. setigera* specimens grown in the ISV were maintained in a greenhouse. To propagate the infected plants, rooted stolons were removed and re-potted about once a year. This process of maintenance was the same at ISV, IVV, and CIRAD. It shall be noted here that the main goal of propagating DSV-infected *D. setigera* at the ISV was the mass production of infected leaf material for virus particle purification suitable for X-ray crystallography. Hence, no particular focus was then on maintaining particular (constant) growth conditions.

In the first articles published on DSV, the natural host plant for this virus was thought to be *Digitaria sanguinalis* (Dollet et al., 1986; Donson et al., 1987; Julia and Dollet 1989). However, this plant is not native to Vanuatu, where the original DSV-infected plant was collected. At the ISV, the chloroplast gene for NADH dehydrogenase subunit F of the plant sampled was compared with that of a *D. sanguinalis* specimen of a field near the ISV and with that of the *D. setigera* (GenBank accession number AY029629.1). The analysis showed that the specimen collected from Vanuatu and grown at CIRAD, IVV, and ISV belonged to the species *D. setigera*, the natural host of DSV, and not to *D. sanguinalis*. For the amplification of NADH dehydrogenase F genes, two PCR primers were used

DSNADHF1819R (5ʹ-TGCTATGATAGACCAAAAATTGC-3ʹ) and DSNADHF1416F (5ʹ-GGGGAAAAGGCATATCCAAA-3ʹ).

Between 1990 and 2010, fifteen viral DNA samples were collected and stored at −80°C (Table 1). Most of the samples were extracted from leaves of specimens stored in the ISV. In this centre, new *D. setigera* specimens were obtained in 2002 through vegetative propagation of the original one. The resulting plants were grown in separate pots. In one of these pots, the viral sample ISV 2008-A was obtained. The other specimen served as a mother plant for the obtention of two new plants in 2007, separated in two different pots. Samples ISV 2008-B, ISV 2008-B1, ISV 2008-B1-FTA, and ISV 2008-B2 were extracted from the same specimen, while the sample ISV 2008-C was obtained of the other B-lineage plant (Fig. 1). The ISV 2008-B1 and ISV 2008-B1-FTA samples were obtained from the same leaf using different extraction methods, as were the IVV 2008 and IVV 2008-FTA samples, obtained from the specimen located in the IVV (Italy) in 2008, generously provided by G. P. Accotto. Finally, the CIRAD 2010 sample was extracted from a specimen grown at CIRAD, kindly provided by M. Peterschmitt in 2010. Thus, in total six *D. setigera* specimens served for DSV sampling in our study (Fig. 1).

**Table 1.** List of DSV samples by origin and date of sampling.

Sample label	Lineage, see Fig. 1	Date of sampling	Site of sampling
ISV 1990^a^	3	16 January 1990	ISV (CNRS, Gif-sur-Yvette, France)
ISV 1998^a^	3	17 November 1998	ISV (CNRS, Gif-sur-Yvette, France)
ISV 1999-01	3	30 January 1999	ISV (CNRS, Gif-sur-Yvette, France)
ISV 1999-07	3	03 July 1999	ISV (CNRS, Gif-sur-Yvette, France)
ISV 1999-12	3	08 December 1999	ISV (CNRS, Gif-sur-Yvette, France)
ISV 2001^a^	3	11 May 2001	ISV (CNRS, Gif-sur-Yvette, France)
ISV 2008-A^b^	4	10 June 2008	ISV (CNRS, Gif-sur-Yvette, France)
ISV-2008-B	5	24 June 2008	ISV (CNRS, Gif-sur-Yvette, France)
ISV 2008-B1^a^	5	10 June 2008	ISV (CNRS, Gif-sur-Yvette, France)
ISV 2008-B1-FTA^a^	5	10 June 2008	ISV (CNRS, Gif-sur-Yvette, France)
ISV 2008-B2^a^	5	10 June 2008	ISV (CNRS, Gif-sur-Yvette, France)
ISV 2008-C^c^	6	10 June 2008	ISV (CNRS, Gif-sur-Yvette, France)
IVV 2008^a^	2	17 June 2008	IVV (Torino, Italy)
IVV 2008-FTA	2	17 June 2008	IVV (Torino, Italy)
CIRAD 2010^a^	1	13 January 2010	CIRAD (Montpellier, France)

a Samples that were analysed for quasispecies complexity (Supplementary Table S3) and the consensus sequences of which were obtained by alignment of molecular clones.

b The ISV 2008-A sample was extracted from a *D. setigera* specimen that was cultivated since 2002, different from the two plants from where the other viral samples were extracted in 2008 (see Fig. 1).

c The ISV 2008-C sample was extracted from a *D. setigera* specimen that was cultivated since 2007, different from the plant from which the ISV 2008-B, ISV 2008-B1, ISV 2008-B1-FTA, and ISV 2008-B2 viral samples were extracted in 2008 (see Fig. 1).

### 2.2 DNA extraction, viral amplification, cloning, and sequencing

For most samples, DNA was extracted by Edwards’ method (Edwards, Johnstone, and Thompson 1991) with modifications (Sánchez-Campos et al., 2018) and amplified by RCA, using the TempliPhi kit (GE Healthcare, Chicago, USA) (Mabvakure et al., 2016). For molecular cloning, RCA products were digested with *Stu*I (Fermentas), which cuts the DSV genome at position 1589. The resulting linear DSV DNA (2.7 kb) was purified with the QIAquick gel extraction kit (Qiagen NV, Hilden, Germany) ligated to EcoRV-linearized pBluescript II KS (±) vector purified the same way (Wu et al., 2008), and introduced into *Escherichia coli* DH5α. Finally, DNA of around twenty cloned recombinant plasmids per sample was amplified by RCA prior to sequencing. For samples where only consensus sequences were determined, ligation reactions were amplified by RCA prior to sequencing. RCA DNAs were sent to GATC Biotech (Constance, Germany), for Sanger sequencing, using the following primers: DSV667R (5ʹ-CTGGGTTGTGCGTCATACAC-3ʹ), DSV2021F (5ʹ-CTCTCCCCAAGAAATGGTGA-3ʹ), M13FP (5ʹ-CCTCTGGCCCAAGTAGACTT-3ʹ), and M13RP (5ʹ-GCCTAGGTAGACATAATTAC-3ʹ).

The reads generated by the reverse primer DSV667R covered part of LIR (2550–2701), the entire *MP* gene (1–330), and part of the *CP* gene (up to position 640). Reads generated by the forward primer DSV2021F covered part of the *RepA* gene (2069–2385), LIR (2386–2701), the *MP* gene, and part of *CP* (up to position 510–515). The reads generated by the forward primer M13FP covered part of *Rep*/*RepA* (position 1590) to part of LIR (position 2550). Finally, the reverse primer M13RP covered part of the *CP* gene and *Rep*/*RepA* (630–1590).

Some viral samples were obtained using Whatman FTA cards (Owor et al., 2007). Each card was impregnated with plant tissue from *D. setigera* leaves. Then a 2.5-mm-diameter fragment from the card was removed with a Harris punch, washed with the washing solution, and left to dry. The piece of card was used directly in 10-μl RCA reaction (Shepherd et al., 2008). DNA product was cloned and sequenced as above. The experimental error frequency of our amplification and cloning system had been determined in Grigoras et al. (2010) and was about 2 orders of magnitude below the observed mutation frequency.

### 2.3 Consensus sequences analysis and evolutionary rate estimation

Consensus sequences (>50 per cent majority rule) were obtained by aligning the sequences of molecular clones of each sample (representing a spectrum of mutants) or by aligning the sequences of the RC-amplified ligation products (for samples in which no molecular cloning was performed) using Seqman and MegAlign (DNASTAR, Lasergene Inc., Madison, WI, USA). The first DSV consensus sequence, published in 1987 (M23022.1), was obtained from GenBank. Nucleotide sequence coordinates used here are as in M23022.1 and do not follow the geminivirus nucleotide numbering convention.

The evolutionary rate or substitution rate was estimated by calculating the number of mutations in the consensus sequence, divided by the total number of nucleotides and by unit of time (years) (Duffy, Shackelton, and Holmes 2008). We used two different evolutionary rate estimates to infer the DSV evolution in highly variable time intervals, from several months to 20** **years. First, we identified polymorphisms among consensus sequences of viral samples obtained in a period between each sampling in the same plant, as well as among different plants obtained by vegetative propagation. Later, we identified polymorphisms present in the consensus sequences obtained of each sample with respect to the sequence registered in 1987 (Donson et al., 1987).

### 2.4 Heterogeneity and evolution of DSV over 20 years

To characterize the genetic complexity of DSV, the composition of the mutant spectrum and the heterogeneity of the viral genomes were analysed in samples ISV 1990, ISV 1998, ISV 2001, ISV 2008-B1, ISV 2008-B1-FTA, ISV 2008-B2, IVV 2008, and CIRAD 2010. To this end, the sequences of molecular clones of each sample were aligned using MUSCLE. Mutations (including base substitutions and InDels) in a mutant spectrum that were not manifested in the consensus sequences were counted. Identical mutations at the same position for each mutant spectrum were counted only once, assuming the same mutations were derived by the replication of a single change event (Domingo et al., 2006).

The mutation frequency was estimated by dividing the number of mutations by the total number of nucleotides sequenced in that sample. Possible biases in nucleotide substitutions and in the distribution of mutations of viral populations as a whole were examined. For this, the weight of each substitution base in percentage and its expected frequency were calculated, assuming that all types of substitutions are equally likely. Qualitative variations were analysed using a 2 × 2 chi-square test (number of observed and expected base substitutions of a particular type × total number of observed and expected base substitutions of the other types), considering a value of *P* < 0.05 as statistically significant (van der Walt et al., 2008; Grigoras et al., 2010). Bias in the distribution of mutations was analysed by calculating the percentage and the statistical value for coding and non-coding sequences. The statistical significance was inferred by obtaining the frequency of expected mutations in the coding and non-coding sequences, considering that they represent 82 per cent and 18 per cent of the DSV genome, respectively. In addition to base substitutions, deletions and insertions (InDels) were identified and annotated. Finally, we compiled all genetic variations (base substitutions and InDels) observed in the mutant spectra for each genomic region.

The complexity of the viral populations was calculated by means of the mean genetic distance (*d*). The *d* value is the average number of mutations per site among any pair of sequences chosen at random from the population. The *d* values were estimated for the entire genome and for each genomic region using the Kimura two-parameter method with MEGA X (Kumar et al., 2018).

To calculate the heterogeneity of the mutant spectra, the normalized Shannon index (Sn) was used, with values ranging from 0 (total homogeneity in the sample) to 1 (all sequences are unique in the sample). For this, we obtained the frequency of each sequence in each population of aligned sequences (Volkenstein 1994).

It should be noted that some mutations were present in more than one sample. Since these are samples from the same plant or lineage, we have considered that these mutations have remained since we first detected them in the mutant spectra over time. In our analyses, for certain parameters (mutation frequency, number of total mutation types for estimating substitution biases, and variability per genomic region) any mutation was counted only once, when it appeared in the chronologically oldest sample. For the estimation of complexity (mean genetic distance) and diversity (Shannon index) of each mutant spectrum, all mutations were considered, including repeats.

### 2.5 Selection pressure

To estimate the selection pressure experienced by the DSV sequences, the dN/dS ratio was calculated. The average dN/dS ratio of all isolated genomes and consensus sequences was calculated for each Open Reading Frame (ORF) (*V1*, *V2*, *C1*, and *C2*). The ratio is obtained by dividing the observed number of non-synonymous (Nd) and synonymous (Sd) substitutions in the virus genomes by the expected number of non-synonymous (ndi) and synonymous (sdi) sites for each codon where mutations were produced (Nei and Gojobori 1986). Using these values, the number of non-synonymous substitutions per non-synonymous site (dN) and the number of synonymous substitutions per synonymous site (dS) were obtained. For this analysis in the mutant spectra, we considered all mutations, including repeats. Potential sites under positive and negative selection in the ORFs were identified using four distinct statistical methods implemented in the DataMonkey webserver (https://www.datamonkey.org/): Mixed Effects Model of Evolution (Murrell et al., 2012), Single Likelihood Ancestor Counting (SLAC), Fixed Effects Likelihood (FEL) (Kosakovsky Pond and Frost 2005) and Fast, Unconstrained Bayesian AppRoximation (FUBAR) (Murrel et al., 2013). The mean ratios of non-synonymous to synonymous substitutions (dN/dS) were estimated for each coding sequence using the SLAC method. All methods were applied using nucleotide substitution models with the best fit for each data set determined in the DataMonkey webserver.

### 2.6 Detection of recombinants

The identification of possible recombinant sequences in each mutant spectrum was carried out using the RDP4 program, employing the methods included in its configuration: RDP, GeneConv, MaxChi, Chimaera, BootScan, SiScan, and 3seq. Recombination events detected by at least three different methods were considered. An estimation of the confidence intervals of the recombination breakpoint was calculated (Martin et al., 2015).

### 2.7 Phylogenetic analysis

The fifteen DSV consensus sequences plus the Donson et al.’s (1987) DSV sequence (GenBank Accession M23022.1) were aligned in MEGA X (Kumar et al., 2018) using MUSCLE. First, a model test was carried out in MEGA X to find the best nucleotide substitution model fitting for the data (Kumar et al., 2018). It was determined that the best surrogate models for phylogenetic tree construction were Jukes–Cantor and Kimura two-parameter models. Phylogenetic analysis was performed on multiple sequence alignments, using the maximum likelihood method under the Kimura two-parameter model with 1,000 bootstrap replications, as implemented in MEGA X (Kumar et al., 2018). The sequences were aligned in MUSCLE format. A phylogenetic tree was built from the alignment of the consensus sequences. The trees have been deposited in the TreeBASE database (Submission ID: 28769, URL: http://purl.org/phylo/treebase/phylows/study/TB2:S28769).

## 3. Results

### 3.1 Twenty years of DSV evolution

**Figure 2.**
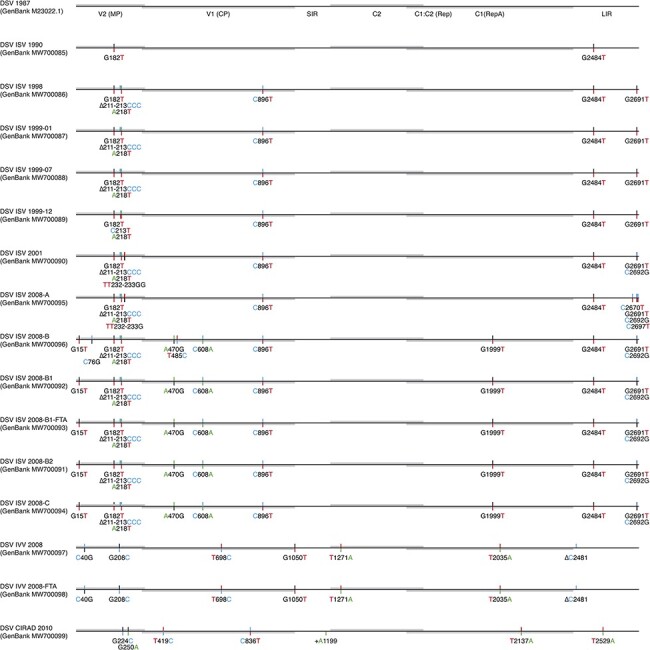
Summary of variations in the consensus sequences of the propagated DSV populations in comparison with the original DSV sequence of 1987 (M23022.1). Consensus sequences are shown in a linear fashion as scaled line drawings, with ORF sequences represented as grey bars. ORF and gene designations are according to Brown et al. (2012). Positions of variable sites are indicated by bicoloured vertical lines. The upper parts of the vertical lines denote the nucleotide present in the sequence of 1987 (M23022.1). The lower parts of the vertical lines denote the new nucleotide detected in the consensus sequences of a given sample. Nucleotides are colour coded as follows: A, green; T, red; C, blue; and G, black. Deletions are represented by (Δ). Insertions are marked by a +.

**Figure 3.**
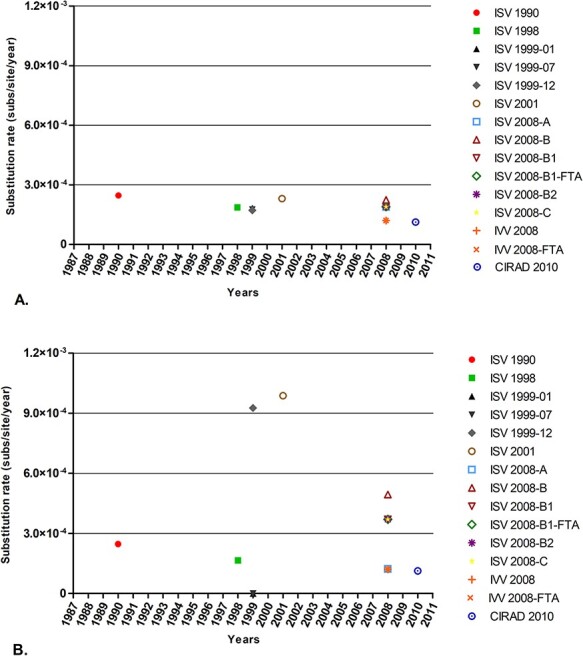
Evolution of DSV populations isolated in *D. setigera*. (A) Nucleotide substitution rates of DSV, which were obtained comparing the changes in the consensus sequences with respect to the consensus sequence published in 1987. (B) Nucleotide substitution rates of DSV that were obtained by comparing changes in each consensus sequence with respect to the consensus sequence established for a previous sample in the same (ISV) plant lineage. Samples ISV 1990, IVV 2008, IVV-2008-FTA, and CIRAD 2010 were compared to the 1987 published sequence.

The evolution of three isolated DSV populations in France and Italy for over two decades was analysed, and their respective rates were determined. Consensus sequences of fifteen samples from six plants were obtained by Sanger sequencing (Fig. 2). Compared to the sequence published in 1987, thirty-one mutations were observed in the consensus sequences sampled over 20 years (Fig. 2). We estimated that DSV evolved within the ranges of 10^−4^ and 10^−3^ subs/site/year, with different ranges determined according to the estimation strategy (Fig. 3A and Supplementary Table S1).

Using the sequence published in 1987 as the reference, we estimated that the nucleotide substitution rate of DSV varied between 1.13 × 10^−4^ and 2.47 × 10^−4^ subs/site/year (Fig. 3A). For 20 years, the evolutionary rate was determined between 1.13 × 10^−4^ and 2.25 × 10^−4^ subs/site/year. The evolutionary rate calculated in the ISV samples was higher (1.72 × 10^−4^–2.47 × 10^−4^ mut/site/year) than that obtained in the samples IVV 2008 and IVV 2008-FTA from Torino (1.21 × 10^−4^ subs/site/year) and sample CIRAD 2010 from Montpellier (1.13 × 10^−4^ subs/site/year) (Supplementary Table S1). *MP* and LIR were the regions that evolved most rapidly (0.85 × 10^−3^ to 1.01 × 10^−3^ and 0.15 × 10^−3^ to 1.06 × 10^−3^ subs/site/year, respectively). In contrast, the *CP* (0.64 × 10^−4^ to 1.91 × 10^−4^ subs/site/year) and *C1*/*C2* (3.48 × 10^−5^ to 7.49 × 10^−5^ subs/site/year) regions were relatively highly conserved (Supplementary Table S1). In the consensus sequences of the ISV samples, the SIR region remained stable, without changes for 21 years (Fig. 2 and Supplementary Table S1).

Focusing on the substitutions in the ISV lineages, we observed that some mutations present in the mutant spectra since the 1990s took time to become fixed in the populations (Fig. 2, Supplementary Tables S1 and S3). This happened, for example, with the non-synonymous mutation G1999T leading to His129Gln, in *RepA*. This polymorphism appeared in the mutant spectrum of 1990 and was not fixed until the samples were isolated in 2008. However, mutations in the *MP* region quickly became predominant (Fig. 2, Supplementary Tables S1 and S3).

When based on the period between each sampling, we estimated that the substitution rate in DSV varied between 1.13 × 10^−4^ subs/site/year and 9.87 × 10^−4^ subs/site/year (Fig. 3B). Estimates of rates of DSV evolution were impacted by the timescale of measurement. The estimated evolutionary rates in short intervals of time (less than 1 year) were pronouncedly greater than the evolutionary rates estimated in intervals of several years, up to an order of magnitude (Fig. 3B). The highest evolution rate estimates (approximately 10^−3^ subs/site/year) were recorded at very short time intervals: between the consensus sequence of the sample ISV 1999-12 (almost 5 months) and in the consensus sequence obtained of the sample ISV 2001 (18 months) (Fig. 3B, Supplementary Table S1). The dispersion in the evolution rates was caused by changes in the *MP* region (Supplementary Table S1). In this region, the nucleotide substitution rate was considerably higher than the genome means. From 1999, the rate of substitution in the *MP* region of the consensus sequences obtained from the ISV was estimated to be within the range of 10^−3^ subs/site/year (1.52–7.58 × 10^−3^ subs/site/year).

### 3.2 Quasispecies complexity and heterogeneity analyses

**Figure 4.**
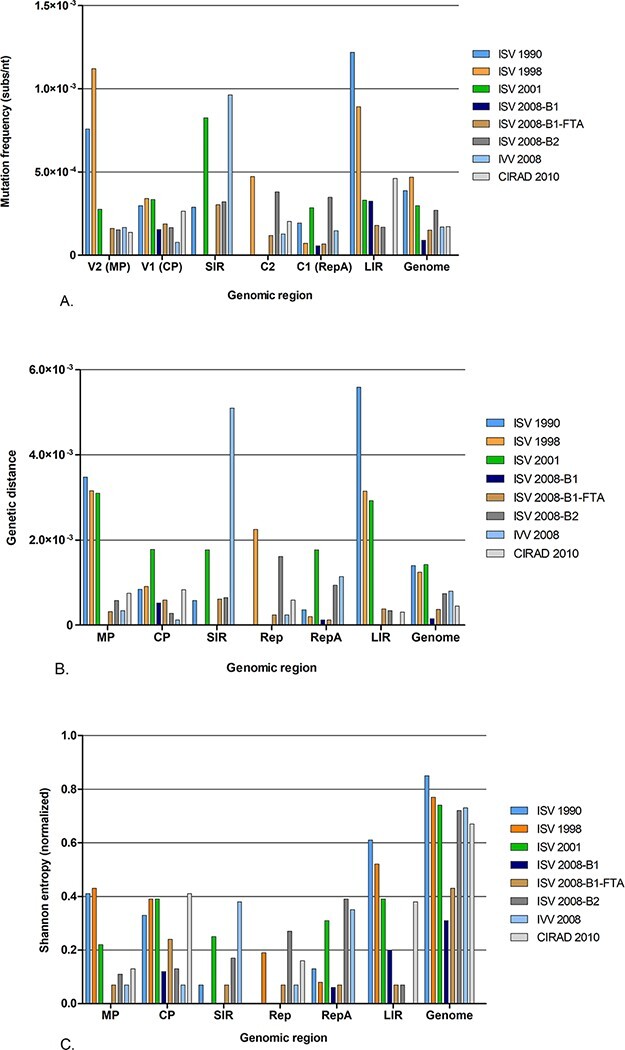
Genetic complexity and haplotype diversity of DSV whole-genome sequences and genomic region. (A) Mutant frequency. (B) Genetic distance. (C) Normalized Shannon entropy.

An in-depth genetic characterization of the viral populations was carried out for eight samples (Table 2). A total of 412,853 nucleotides representing 165 partial genomes were sequenced, in which 99 mutations were identified. A rapid and constant generation of mutant spectra was observed, both during infections maintained for years and after vegetative propagation (Fig. 4, Supplementary Tables S2–S6 and Table 2).

**Table 2.** Estimation of complexity and heterogeneity parameters for eight of the DSV samples isolated in this study.

Samples	Mutations^a^^b^	Nucleotide sequence	Complexity and diversity of DSV populations		
			Mutation frequency (mut/nt)	Genetic distance	Shannon entropy
ISV 1990	20	53,254	3.88 × 10^−4^	1.40 × 10^−3^	0.85
ISV 1998	20	46,074	4.69 × 10^−4^	1.49 × 10^−3^	0.83
ISV 2001	16	51,064	2.98 × 10^−4^	1.42 × 10^−3^	0.75
ISV 2008-B1	5	54,827	9.05 × 10^−5^	1.50 × 10^−4^	0.31
ISV 2008-B1-FTA	7	48,784	1.51 × 10^−4^	3.70 × 10^−4^	0.43
ISV 2008-B2	13	49,834	2.70 × 10^−4^	7.40 × 10^−4^	0.72
IVV 2008	8	48,788	1.70 × 10^−4^	8.00 × 10^−4^	0.73
CIRAD 2010	10	59,958	1.73 × 10^−4^	4.50 × 10^−4^	0.67

a Each mutation identified at one position in each mutant spectrum was counted only once, whether it was present in one or more genomes of the quasispecies.

b Mutations present in more than one sample have only been counted once, in the chronologically oldest samples.

The detection of point mutation biases was statistically validated by means of the *P*-value using a 2 × 2 chi-square test (Supplementary Table S2). The existence of biases in base substitutions was observed in the set of the mutant spectra, related to G/C→A/T mutations. We found that C→T transitions and G→T transversions were overrepresented. Mutation biases compatible with G→T transversions and C→T transitions would lead to a decrease in cytosine and guanine content. Samples showed a variation of G/C→A/T substitutions that significantly decreased the GC content (*P* < 0.05) (Supplementary Table S2). On the other hand, no underrepresentation in A/T→G/C substitutions was observed in all samples (*P* > 0.05). A statistically significant underrepresentation of A→C and G→C transversions in the samples (*P* < 0.05) was observed (Supplementary Table S2). G/C→A/T mutations led to a higher abundance of transition events (Ts/Tv > 0.5). However, the Ts/Tv values were not high enough for a significant trend towards transitions in the mutant spectra (Supplementary Table S2).

InDels (insertions and deletions) were common in the DSV genomes. The relative abundance of InDels in the mutant spectra was variable (Supplementary Table S2). InDels were found mostly in the non-coding sequences of the genome.

The search for distribution of mutation biases was performed by noting the total number of mutations observed in the coding regions (*MP*, *CP*, *C2*, and *RepA* or *C1*) and in the intergenic regions (LIR and SIR) of all mutant spectra (Supplementary Table S2). Based on a chi-square distribution test, we observed an uneven distribution of genetic variations in the analysed sequences (Supplementary Table S2). The non-coding intergenic regions had a high mutation frequency mainly due to the abundance of InDels (*P* < 0.05). On the other hand, the number of mutations observed in the coding regions in all samples was lower than expected but not statistically significant (*P* > 0.05) (Supplementary Table S2).

The complexity and heterogeneity of the DSV quasispecies were evaluated by mutation frequency, mean genetic distance, and normalized Shannon entropy. For this purpose, we analysed the variations present in the spectra of mutants at the complete genome level (Table 2) and for each genomic region (Supplementary Table S4).

The mutation frequency measures the proportion of mutations in a spectrum of mutants with respect to its consensus sequence. Estimated mutation frequencies ranged from 9.05 × 10^−5^ to 4.69 × 10^−4^ mut/nt. We did not observe significant differences in the estimated mutation frequencies for the mutant spectra (Table 2). However, there were strong differences in each genomic region. Considering the overall variability in each genomic region, the ORFs *C1* (*RepA*) and *C2* were the least variable (1.47 and 1.63 × 10^−4^ mut/nt, respectively) (Supplementary Table S4). On the contrary, the *MP* gene and the non-coding SIR and LIR sequences were the most variable regions (Fig. 4A, Supplementary Table S4).

With the mean genetic distance (*d*), we measured the mean value of the mutations existing between pairs of randomly chosen sequences of the population. In the ISV 1990, ISV 1998, and ISV 2001 samples, we observed a higher value of *d* than in the other samples (Table 2). The *MP* and LIR regions showed a greater genetic complexity than any other regions (Supplementary Table S4). The *d* values varied for each sample, especially in the SIR that in many samples remained invariant, whereas in the sample IVV 2008, a high value of *d* was observed (Fig. 4B, Supplementary Table S4). A significant decrease in mutation frequencies and genetic distance was observed in the ISV samples of 2008 (specifically in ISV 2008-B1 and ISV 2008-B1-FTA), compared to the first samples of ISV mutant spectra (ISV 1990, 1998, and 2001). This decrease in genetic complexity was more pronounced in the *MP* and LIR regions. Genetic complexity in *MP* was also significantly lower in the IVV 2008 and CIRAD 2010 samples than in the first samples of the ISV lineage (Fig. 4B, Supplementary Table S4).

The heterogeneity of mutant spectra was evaluated using the normalized Shannon entropy (Sn), which measures the uniqueness and frequency of a sequence in a population. For the whole genome, the Shannon entropy ranged from 0.31 to 0.85 (Table 2). Except for ISV 2008-B1 and ISV 2008-B1-FTA that showed Sn < 0.5, the mutant spectra of other samples showed high heterogeneity (Table 2 and Fig. 4C). Heterogeneity varied according to genomic region. The most heterogeneous regions in respect to the mutant spectra were the *CP* gene and the non-coding sequence LIR (up to a value of 0.6) (Supplementary Table S4).

### 3.3 Selection pressure

In the consensus sequences of all fifteen DSV samples, thirteen non-synonymous mutations and ten synonymous or silent mutations were identified in individual ORFs (Supplementary Table S5). The average ratio of non-synonymous substitutions per non-synonymous site (dN) and synonyms substitutions per synonymous site (dS), dN/dS, was calculated. Adaptive changes by positive and negative selection were detected. When analysing individual ORFs, we observed a high negative selection in the mutations fixed in the consensus sequences for 20 years (Supplementary Table S5). In reference to the 1987 sequence, all the consensus mutations observed in the *CP* regions were synonymous, and the *C1* (*RepA*) and *C2* regions experienced a strong purifying selection (Supplementary Table S5). A value of dN/dS > 1 would indicate the probability that some ORF could undergo positive selection pressure. The *MP* consensus had a value of dN/dS = 6.130, indicating that the viruses experienced a strong positive selection that favoured the fixation of mutations in this gene (Supplementary Table S5). Only the *MP* regions experienced positive selection, where all mutations fixed in the consensus sequences were non-synonymous except the transition C213T (Supplementary Table S1).

Concerning the analyses of mutant spectra of 8 DSV samples indicated in Table 2, forty-three non-synonymous mutations and twenty-six synonymous mutations were identified (Supplementary Table S5). In the *MP* gene, the dN/dS ratio was 4.994, suggesting that the mutant spectra of this region underwent a considerably strong positive or diversifying selection. In the mutant spectra, *C2* and *C1* (*RepA*) experienced different selection pressure but closer to the value of 1 (1.227 and 0.844, respectively). In contrast, the *CP* region experienced a strong negative or purifying selection (dN/dS = 0.167) (Supplementary Table S5).

Applying statistical analyses, evidence of positive diversifying selection on non-synonymous sites (dN/dS > 1) was detected in only three codons of the *MP* and *CP* regions (Supplementary Table S6). Based on this analysis, these three sites appear to evolve under diversifying selection. Interestingly, all sites detected under positive selection were found only in the ISV phylogenetic group. In *MP*, despite detecting many non-synonymous sites, only the change at codon 78 was statistically supported (Supplementary Table S6). The two changes (Ser > Leu and Gly > Leu) at codon 78 were the only alterations fixed in the consensus sequences (of samples ISV 2001 and ISV 2008-A).

Significant purifying (negative) selection was observed in six of the sixteen codons that evolved to dN/dS < 1 using the FEL (six codons), SLAC (one codon), and FUBAR (six codons) methods; the codons were in the *CP*, *RepA*, and *C2* genes (Supplementary Table S6). No sites under purifying selection validated by any of the three methods were detected in the *MP* gene. Furthermore, the negative selection of codon 115 position of *CP* gene was the only one statistically supported by three methods.

### 3.4 Detection of recombinants

Two recombinant viruses were detected in the mutant spectra by sequence analysis of each clone using the RDP4 program. These exchanges of genetic material occurred by intraspecific recombination and were validated by four different methods in the software (Supplementary Table S7).

Focusing on the mutant spectrum in the ISV 1990 sample, a peculiar sequence was detected between the recombination breakpoints of clone 10 (Supplementary Table S7). The alignment of the sequences of the mutant spectrum showed that this sequence ranged from position 1111 to position 1207 in the SIR region of the viral genome. BLASTn analysis showed it was a sequence from the *C1* (*RepA*) and *C2* ORFs from position 1686 to position 1590. Also, in haplotype 16 of the ISV 1998 sample, another very different sequence was detected, ranging from position 1896 to position 2180 in the *RepA* region (Supplementary Table S7). It was identified by BLASTn analysis as a sequence of *C1* (*RepA*) and *C2* ORFs positions 1590–1844.

### 3.5 Phylogenetic analysis

**Figure 5.**
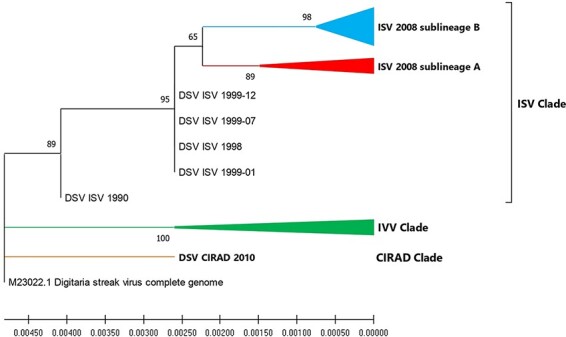
Phylogenetic analysis of DSV consensus sequences. For the phylogenetic reconstruction, the *Maximum Likelihood* method was used with a bootstrap of 1,000 replicates implemented in the MEGA X program. The tree was rooted using the DSV sequence of 1987 (GenBank M23022.1). Branches with low bootstrap support (<50 per cent) were collapsed. Sublineage B groups the consensus sequences of samples extracted from plants 5 (ISV 2008-B, ISV 2008-B1, ISV 2008-B1-FTA, ISV 2008-B2) and 6 (ISV 2008-C), while sublineage A groups the ISV 2001 and ISV 2008-A sequences. The IVV lineage groups the sequences of IVV samples (IVV 2008 and IVV 2008-FTA). See also Fig. 1. The bar below the tree indicates nucleotide substitutions per site.

Phylogenetic analyses allowed inferring the evolution of DSV during the years of propagation in *D. setigera* plants (Fig. 5). We used the sequence deposited in the GenBank database by Donson et al. (M23022.1), as the ancestral sequence. Although the consensus sequences obtained were highly similar (all sequences come from the same DSV lineage), we observed a branching of the sequences within the family tree depending on the centre from which they were obtained. According to the results obtained, there were three well-defined clades within the tree, each representing the sequences of each facility: ISV, IVV, and CIRAD. Apparently, genetic bottlenecks caused by vegetative propagation led to a divergence of virus populations at IVV and ISV from the original plant virus population maintained at CIRAD (Fig. 5). The effect of bottlenecks on the diversification of viral populations could be seen in more detail in the branch of the ISV consensus sequences (Fig. 5). In 2002 and 2007, propagation of the *D. setigera* specimen at the ISV was augmented, which may have increased the diversification of the virus population and the consolidation of a new DSV lineage. Additionally, the branching of the ISV branch from the ISV 2001 sequence (Fig. 5) suggested a possible diversification in sublineages within the ‘ISV-clade’. The consensus sequence ISV 2008-A was distant from the other sequences obtained that year in the ISV samples. The alignment of sequences revealed that this consensus sequence differed in eight bases from the other sequences (Supplementary Table S1).

## 4. Discussion

In the present study, we addressed the evolution of the mastrevirus DSV infecting the grass *D. setigera*. We analysed plant specimens maintained by vegetative propagation over a period of 20 years in three separate laboratories starting from the same initial infected plant. We studied the genetic distance and population genetic variability of DSV samples taken from six *D. setigera* specimens. We focused on the analysis of almost complete individual viral genomes to study evolutionary rates as well as the complexity and diversity of intra-host DSV populations and their phylogenetic relationship.

Using as reference the initial 1987 sequence (Donson et al., 1987), we have obtained substitution rates for DSV estimated between 1.13 × 10^−4^ and 2.47 × 10^−4^ subs/site/year. Estimating the evolution rate for the period between samples extracted of the same plants, we showed that DSV evolved at rates between 1.13 × 10^−4^ and 9.87 × 10^−4^ subs/site/year. These substitution rates are within the range observed in other geminiviruses such as MSV (Isnard et al., 1998; van der Walt et al., 2008; Harkins et al., 2009) and tomato yellow leaf curl virus (Duffy and Holmes 2008; Yang et al., 2014; Mabvakure et al., 2016) and in other ssDNA plant viruses such as faba bean necrotic stunt virus (Grigoras et al., 2010). The evolution rate of DSV and other geminiviruses is within the range of RNA viruses, whose evolution rates vary between 10^−5^ and 10^−2^ subs/site/year (Silva, Marques, and Nolasco 2012; Dapp et al., 2017; Guan et al., 2018; Jones et al., 2019; Su et al., 2020). Despite the fact that ssDNA viruses use less error-prone DNA polymerases than the RNA-dependent RNA polymerases of RNA viruses (Drake 1991; Jenkins et al., 2002; Hicks and Duffy 2014; Wu et al., 2020), they experience evolution rates rivalling those of the most variable RNA viruses. Several factors are responsible for the elevated substitution rates of ssDNA viruses, and from the results of this study, we suggest which factors determined the genetic variability and evolution of DSV quasispecies.

Most of the mutant spectra experienced an overrepresentation of transversions G→T. These results showed evidence that oxidation of guanine to 8-oxoguanine (8-oxodG) could be a key factor in the genetic variability of DSV genomes, as it was also reported for MSV (van der Walt et al., 2008). We also observed substitution biases towards transitions by deamination (C→T), processes that have been studied in geminiviruses and nanoviruses (Duffy and Holmes 2008; Grigoras et al., 2010). ssDNA molecules can suffer oxidative stresses, generated by spontaneous or host enzyme–induced deamination and oxidation processes (Duffy, Shackelton, and Holmes 2008).

Deaminase enzymes constitute an antiviral defence mechanism that increases the production of deleterious mutations, a process known as lethal mutagenesis or entry in error catastrophe (Domingo and Perales 2019). In humans, the deaminase APOBEC3G is an innate inhibitor of retroviruses and endogenous retroviral elements (Miyagi et al., 2007; Okada and Iwatani 2016), as well as dsDNA viruses (Hirose et al., 2018). Plant enzymes that introduce G→A transitions in the reverse transcription step of caulimovirus replication have been reported (Martín et al., 2017). The deaminase activity of APOBEC3G does not contribute to genetic variability in retroviruses as much as reverse transcriptase does (Delviks-Frankenberry et al., 2016). However, ssDNA viruses use high-fidelity DNA polymerases (Drake 1991; Wu et al., 2020), and therefore, the activity of these enzymes may be relevant for the generation of genetic diversity in DSV and in agronomically important geminiviruses. In humans, APOBEC3G can deaminate cytosines in ssDNA for virus restriction (McDaniel et al., 2020). However, more extensive testing is needed to support this hypothesis.

Emerging ssDNA viruses from plants and some ssDNA viruses from animals were found to be organized as quasispecies (Correa-Fiz et al., 2018; Sánchez-Campos et al., 2018; Juárez et al., 2019; Van Loy et al., 2015), as amply reported for RNA viruses and reviewed by Domingo, Sheldon, and Perales (2012). The variability and heterogeneity of the DSV populations studied here corroborate that also this virus is organized as quasispecies. In our study, bottlenecks were produced by vegetative propagation of *D. setigera*. After characterizing the mutant spectra, we confirmed that sequences fixed as consensus reflected only a small part of the genetic variability that the viruses experienced, whereas in the *MP* gene, the mutations established as consensus closely reflected the overall sequence variability. The limitations of consensus sequences have to be considered when studying viral quasispecies. Many mutations and recombination events are a minority and are not reflected in the consensus sequence but may have implications for viral pathogenesis and evolution (Domingo 2016; Domingo and Perales 2019). Experimental studies have reported that minority genomes within a spectrum of RNA virus mutants may include mutations that confer selective advantage over antiviral therapies (Moreno et al., 2017; Donohue, Pfaller, and Cattaneo 2019). It has been reported that diversification of mutant spectra from the same strain in environments subject to different selection pressures generates a specific mutational composition without changing the consensus sequence (Sánchez-Campos et al., 2018).

The strong positive selection experienced by the *MP* region promoted the fixation of mutations in the consensus sequences, yet the genetic diversity was very low in the sequences of the 2008 and 2010 samples (Supplementary Table S2, *Variability by regions*). Our results suggest that natural selection favoured the fixation of amino acid changes in the MP, providing an adaptive advantage. MP protein mediates the intra- and intercellular virus movement in the plant (Lefeuvre et al., 2019). Under vegetative propagation (i.e. without vector transmission), bottlenecks generated for instance by producing new symptomatic plantlets from rooted stolon nodes, as well as by the movement of the virus through the plant, constituted a potential means of positive selective pressure on the *MP* region. In the *CP* region, there was greater genetic diversity but with little change in the consensus sequences. The genetic variability of the *CP* in a begomovirus critically impacts the transmission efficiency by different whitefly vectors of the virus in an experimental system (Pan et al., 2020), since the coat proteins interact with the proteins of the vector insect (Lefeuvre et al., 2019). Without virus–vector transmission, the constraint on the *CP* genes of the quasispecies is released, which leads to greater sequence variation but not to the fixation of mutations in the consensus sequences (Sánchez-Campos et al., 2018; Pinto et al., 2021).

Diversifying selection may have favoured the sustained maintenance of DSV within *D. setigera* and the movement and colonization of the new plantlets emerging from rooted stolon nodes over 20 years. No obvious change of symptom severity over time was observed. However, no detailed data were collected to analyse eventual DSV symptom evolution during the 20 years of sampling. A recent study has shown evidence that MSV-A has evolved for 110 years in a way that has increased host colonization capacity while decreasing host damage (Monjane et al., 2020).

Our analyses showed few recombination events. Recombination is a common source of genetic variability that influences the evolution and pathogenicity of geminiviruses (Lefeuvre and Moriones 2015; Kraberger et al., 2017; Rodríguez-Negrete et al., 2019). Recurrent emergence of recombinant geminiviruses has been reported in geographic areas where the parents coexisted naturally or were induced by agriculture (García-Andrés et al., 2006; Díaz-Pendón et al., 2019; Fiallo-Olivé et al., 2019). A study reported that the pathogenic strain MSV-A originated from recombination between the *Digitaria*-adapted strains MSV-B and MSV-F/G viruses (Varsani et al., 2008). However, Lima et al. (2017) reported that mutational dynamics, rather than recombination, is the main driver of geminivirus diversification. Our results seem to be in agreement with this. Maintaining virus populations in confined laboratory or greenhouse environments restricts the chances for genetic exchange with different virus strains or populations via natural insect vector transmission.

In two recombinant viruses (clone 10 ISV 1990 and clone 16 ISV 1998), we observed insertions of intraspecific sequences from other positions of the DSV genome. In ssDNA viruses, recombination events are very frequent, resulting in new chimeric sequences causing new virus species to emerge (Diemer and Stedman 2012; Roux et al., 2013; Krupovic et al., 2015; Kazlauskas et al., 2017; Kazlauskas, Varsani, and Krupovic 2018).

Phylogenetic analyses showed that mutant spectra isolated from DSV samples of *D. setigera* propagated at the ISV diverged from those isolated in the CIRAD, Montpellier, and in the IVV, Torino. As a result, three well-defined phylogenetic groups formed. The reproduction of *D. setigera* by vegetative propagation most probably resulted in bottlenecks that accelerated the diversification of the viruses maintained in the ISV. These processes led to the divergence of a new genetic lineage. In addition, we observed a diversification of the virus population maintained at the ISV after 2002, which lead to the divergence into two sublineages. The genetic drift after a bottleneck, be it by limited vegetative propagation of the host or following transmission between hosts, can induce the emergence of new genetic lineages, which may even cause viral outbreaks (Juárez et al., 2019; Kadoya et al., 2020).

Genetic drift, resulting from founder effects following bottleneck events, could be decisive in the diversification of DSV mutant spectra. The processes of specific selection or stochastic drift following genetic bottlenecks are factors that influence the composition of viral quasispecies (Domingo and Perales 2019). However, genetic drift would not explain the estimated significant differences in dN/dS ratios between the *MP* and *CP* regions. Although they could not be validated in the statistical analyses, there was a trend in *MP* that favoured the emergence and fixation of non-synonymous mutations in the consensus sequences. On the other hand, statistical analyses detected evidence that the codon 78 change, fixed in the ISV 2001 and ISV 2008-A consensus sequences, was under positive selection pressure. Both consensus sequences are in an ISV phylogenetic clade subgroup (sublineage A), distant from the rest of the DSV ISV 2008 sequences (sublineage B). This fact suggests a DSV diversification in the *MP* region under diversifying selection within the ISV lineage.

A selective influence of the environment in which a virus population proliferates must also be considered. There is a potential adaptive evolution that serves as a source of genetic variability for viruses, with multiple routes for fitness gain after a bottleneck (Sánchez-Campos et al., 2018; Donohue, Pfaller, and Cattaneo 2019; Juárez et al., 2019; González et al., 2021). Although we cannot specifically define the variations of the growth and propagation conditions to which the DSV-infected plants were subjected, we can be certain that the environmental conditions were different at the three locations where DSV was maintained. The evolution of viruses and virus–host interaction is affected by the environment the host is in outbreaks (González et al., 2021).

It should be mentioned that we detected two parallel mutations present in samples from infected plants cultivated at different geographical locations. These were the substitution C2023A in *RepA* (seen in ISV 1998 and IVV 2008) and the deletion G2669DEL in LIR (seen in ISV 2001 and CIRAD 2010). These mutations likely have originated independently in individual DSV populations, but we cannot rule out the possibility that the mutants might have remained undetected in ancestral swarms of DSV genomes until their progeny was uncovered in samples from different centres.

In this study, we revealed key aspects of the mutational dynamics and evolution of DSV. We present evidence following the evolution of DSV over about 20 years that the virus evolved as rapidly as other geminiviruses. The evolution rates of the more variable RNA viruses were usually estimated in short-term experiments. The DSV populations in *D. setigera* were organized as quasispecies displaying both high genetic complexity and high heterogeneity in their mutant spectra. The elevated mutation frequencies and the generation of bottlenecks allowed for further diversification of the quasispecies. The genetic variability was not evenly distributed throughout the DSV genome, with *MP* and non-coding regions being the most complex regions. The *MP* gene played a distinctive role in the evolution of the DSV quasispecies, as it underwent diversifying selection in the mutant clouds. The mutation frequency in the *MP* gene was comparably low after 2001, yet the mutations were fixed in the consensus. By contrast, the high number of mutations observed in the *CP* regions of the DSV population did not become fixed in the consensus sequence. Such a striking difference between positive versus negative selection in the movement vs. capsid protein genes of an ssDNA plant virus has never before been described.

## Supplementary Material

veab083_Supp

## Data Availability

GenBank accession numbers for all sequences used in this study are listed in Supplementary Table S8. Data available at doi:10.5061/dryad.vmcvdncs4.
