# Supplementary material for: Twenty years of evolution and diversification of digitaria streak virus in Digitaria setigera
Source: Virus Evol. 2021 Oct 13;7(2):veab083. doi: 10.1093/ve/veab083 (PMC8516820; doi:10.1093/ve/veab083)
Supplement: veab083_Supp [file veab083_supp.zip › Supplementary Table S1_Ortega del Campo_VE.docx]

**Supplementary Table S1.** Mutations and substitution rates of consensus sequences analysed in this study calculated by comparison with either the DSV consensus sequence of 1987 (Donson et al., 1987) or with respect to the previous consensus sequence.

**S1-A.** Mutations of consensus sequences analysed in this study calculated by comparison with the DSV consensus sequence of 1987 (Donson et al., 1987)

| **Genomic region** | **ISV 1990** | **ISV 1998** | **ISV 1999-01** | **ISV 1999-07** | **ISV 1999-12** | **ISV 2001** | **ISV 2008-A** | **ISV 2008-B** | **ISV 2008-B1** | **ISV 2008-B1-FTA** | **ISV 2008-B2** | **ISV 2008-C** | **IVV 2008** | **IVV 2008-FTA** | **CIRAD 2010** |
| --- | --- | --- | --- | --- | --- | --- | --- | --- | --- | --- | --- | --- | --- | --- | --- |
| **V2 (MP)** | G182T G®V | G182T G®V | G182T G®V | G182T G®V | G182T G®V | G182T G®V | G182T G®V | G15T Q®H | G15T Q®H | G15T Q®H | G15T Q®H | G15T Q®H | C40G Q®E | C40G Q®E | G224C R®T |
|  |  | CCC211-213DEL | CCC211-213DEL | CCC211-213DEL | C213T P®P | CCC211-213DEL | CCC211-213DEL | C76G Q®E | G182T G®V | G182T G®V | G182T G®V | G182T G®V | G208C G®R | G208C G®R | G250A V®I |
|  |  | A218T E®V | A218T E®V | A218T E®V | A218T E®V | A218T E®V | A218T E®V | G182T G®V | CCC211-213DEL | CCC211-213DEL | CCC211-213DEL | CCC211-213DEL |  |  |  |
|  |  |  |  |  |  | T232G L®V | T232G L®V | CCC211-213DEL | A218T E®V | A218T E®V | A218T E®V | A218T E®V |  |  |  |
|  |  |  |  |  |  | T233G L®T | T233G L®T | A218T E®V |  |  |  |  |  |  |  |
|  |  |  |  |  |  |  |  |  |  |  |  |  |  |  |  |
| **V1 (CP)** |  | C896T L®L | C896T L®L | C896T L®L | C896T L®L | C896T L®L | C896T L®L | A470G T®T | A470G T®T | A470G T®T | A470G T®T | A470G T®T | T698C F®F | T698C F®F | T419C A®A |
|  |  |  |  |  |  |  |  | T485C T®T | C608A P®P | C608A P®P | C608A P®P | C608A P®P |  |  | C836T S®S |
|  |  |  |  |  |  |  |  | C608A P®P | C896T L®L | C896T L®L | C896T L®L | C896T L®L |  |  |  |
|  |  |  |  |  |  |  |  | C896T L®L |  |  |  |  |  |  |  |
| **SIR** |  |  |  |  |  |  |  |  |  |  |  |  | G1050T | G1050T | INS1199A |
| **C1 (RepA)** |  |  |  |  |  |  |  | G1999T H®Q | G1999T H®Q | G1999T H®Q | G1999T H®Q | G1999T H®Q | T2035A P®P | T2035A P®P | T2137A P®P |
| **C2** |  |  |  |  |  |  |  |  |  |  |  |  | T1271A A®A | T1271A A®A |  |
| **LIR** | G2484T | G2484T | G2484T | G2484T | G2484T | G2484T | G2484T | G2484T | G2484T | G2484T | G2484T | G2484T | C2481DEL | C2481DEL | T2529A |
|  |  | G2691T | G2691T | G2691T | G2691T | G2691T | C2670T | G2691T | G2691T | G2691T | G2691T | G2691T |  |  |  |
|  |  |  |  |  |  | C2692G | G2691T | C2692G | C2692G | C2692G | C2692G | C2692G |  |  |  |
|  |  |  |  |  |  |  | C2692G |  |  |  |  |  |  |  |  |
|  |  |  |  |  |  |  | C2697T |  |  |  |  |  |  |  |  |

**S1-B.** Mutations of consensus sequences analysed in this study calculated by comparison with respect to the previous consensus sequence

| **Genomic region** | **ISV 1990** | **ISV 1998** | **ISV 1999-01** | **ISV 1999-07** | **ISV 1999-12** | **ISV 2001** | **ISV 2008-A** | **ISV 2008-B** | **ISV 2008-B1** | **ISV 2008-B1-FTA** | **ISV 2008-B2** | **ISV 2008-C** | **IVV 2008** | **IVV 2008-FTA** | **CIRAD 2010** |
| --- | --- | --- | --- | --- | --- | --- | --- | --- | --- | --- | --- | --- | --- | --- | --- |
| **V2 (MP)** | G182T G®V | CCC211-213DEL |  |  | INS211-213CCT | CCC211-213DEL |  | G15T Q®H | G15T Q®H | G15T Q®H | G15T Q®H | G15T Q®H | C40G Q®E | C40G Q®E | G224C R®T |
|  |  | A218T E®V |  |  |  | T232G L®V |  | C76G Q®E | G232T G®W | G232T G®W | G232T G®W | G232T G®W | G208C G®R | G208C G®R | G250A V®I |
|  |  |  |  |  |  | T233G L®T |  | G232T G®W | G233T G®V | G233T G®V | G233T G®V | G233T G®V |  |  |  |
|  |  |  |  |  |  |  |  | G233T G®V |  |  |  |  |  |  |  |
|  |  |  |  |  |  |  |  |  |  |  |  |  |  |  |  |
| **V1 (CP)** |  | C896T L®L |  |  |  |  |  | A470G T®T | A470G T®T | A470G T®T | A470G T®T | A470G T®T | T698C F®F | T698C F®F | T419C A®A |
|  |  |  |  |  |  |  |  | T485C T®T | C608A P®P | C608A P®P | C608A P®P | C608A P®P |  |  | C836T S®S |
|  |  |  |  |  |  |  |  | C608A P®P |  |  |  |  |  |  |  |
| **SIR** |  |  |  |  |  |  |  |  |  |  |  |  | G1050T | G1050T | INS1199A |
| **C1 (RepA)** |  |  |  |  |  |  |  | G1999T H®Q | G1999T H®Q | G1999T H®Q | G1999T H®Q | G1999T H®Q | T2035A P®P | T2035A P®P | T2137A P®P |
| **C2** |  |  |  |  |  |  |  |  |  |  |  |  | T1271A A®A | T1271A A®A |  |
| **LIR** | G2484T | G2691T |  |  |  | C2692G | C2670T |  |  |  |  |  | C2481DEL | C2481DEL | T2529A |
|  |  |  |  |  |  |  | C2697T |  |  |  |  |  |  |  |  |

**S1-C.** Substitution rates of consensus sequences analysed in this study calculated by comparison with the DSV consensus sequence of 1987 (Donson et al., 1987)

| **Samples** | **Years** | **Mutations** | **Mut/site** | **Evolution rate (subs/site/year)** | | | | | |
| --- | --- | --- | --- | --- | --- | --- | --- | --- | --- |
|  |  |  |  | **Entire genome** | **V2 (MP)** | **V1 (CP)** | **SIR** | **C2/C1 (RepA)** | **LIR** |
| **ISV 1990** | 3 | 2 | 7.40 × 10^-4^ | 2.47 × 10^-4^ | 1.01 × 10^-3^ | NA | NA | NA | 1.06 × 10^-3^ |
| **ISV 1998** | 11.9 | 6 | 2.22 × 10^-3^ | 1.87 × 10^-4^ | 7.64 × 10^-4^ | 1.14 × 10^-4^ | NA | NA | 2.68 × 10^-4^ |
| **ISV 1999-01** | 12.1 | 6 | 2.22 × 10^-3^ | 1.84 × 10^-4^ | 5.01 × 10^-4^ | NA | NA | NA | NA |
| **ISV 1999-07** | 12.6 | 6 | 2.22 × 10^-3^ | 1.76 × 10^-4^ | NA | NA | NA | NA | NA |
| **ISV 1999-12** | 12.9 | 6 | 2.22 × 10^-3^ | 1.72 × 10^-4^ | 2.35 × 10^-4^ | NA | NA | NA | NA |
| **ISV 2001** | 14.4 | 9 | 3.70 × 10^-3^ | 2.31 × 10^-4^ | 6.31 × 10^-4^ | NA | NA | NA | 2.21 × 10^-4^ |
| **ISV 2008-A** | 21.4 | 11 | 4.07 × 10^-3^ | 1.90 × 10^-4^ | 7.08 × 10^-4^ | NA | NA | NA | 2.98 × 10^-4^ |
| **ISV 2008-B** | 21.4 | 13 | 4.81 × 10^-3^ | 2.25 × 10^-4^ | 8.50 × 10^-4^ | 1.91 × 10^-4^ | NA | 3.74 × 10^-5^ | NA |
| **ISV 2008-B1** | 21.4 | 11 | 4.07 × 10^-3^ | 1.90 × 10^-4^ | 5.66 × 10^-4^ | 1.27 × 10^-4^ | NA | 3.74 × 10^-5^ | NA |
| **ISV 2008-B1-FTA** | 21.4 | 11 | 4.07 × 10^-3^ | 1.90 × 10^-4^ | 5.66 × 10^-4^ | 1.27 × 10^-4^ | NA | 3.74 × 10^-5^ | NA |
| **ISV 2008-B2** | 21.4 | 11 | 4.07 × 10^-3^ | 1.90 × 10^-4^ | 5.66 × 10^-4^ | 1.27 × 10^-4^ | NA | 3.74 × 10^-5^ | NA |
| **ISV 2008-C** | 21.4 | 11 | 4.07 × 10^-3^ | 1.90 × 10^-4^ | 5.66 × 10^-4^ | 1.27 × 10^-4^ | NA | 3.74 × 10^-5^ | NA |
| **IVV 2008** | 21.4 | 7 | 2.59 × 10^-3^ | 1.21 × 10^-4^ | 2.83 × 10^-4^ | 6.36 × 10^-5^ | 2.72 × 10^-4^ | 7.49 × 10^-5^ | 1.49 × 10^-4^ |
| **IVV 2008-FTA** | 21.4 | 7 | 2.59 × 10^-3^ | 1.21 × 10^-4^ | 2.83 × 10^-4^ | 6.36 × 10^-5^ | 2.72 × 10^-4^ | 7.49 × 10^-5^ | 1.49 × 10^-4^ |
| **CIRAD 2010** | 23 | 7 | 2.59 × 10^-3^ | 1.13 × 10^-4^ | 2.64 × 10^-4^ | 1.18 × 10^-4^ | 2.53 × 10^-4^ | 3.48 × 10^-5^ | 1.38 × 10^-4^ |

**S1-D.** Substitution rates of consensus sequences analysed in this study calculated by comparison with respect to the previous consensus sequence.

| **Samples** | **Years** | **Mutations** | **Mut/site** | **Evolution rate (subs/site/year)** | | | | | |
| --- | --- | --- | --- | --- | --- | --- | --- | --- | --- |
|  |  |  |  | **Entire genome** | **V2 (MP)** | **V1 (CP)** | **SIR** | **C2/C1 (RepA)** | **LIR** |
| **ISV 1990** | 3 | 2 | 7.40 × 10^-4^ | 2.47 × 10^-4^ | 1.01 × 10^-3^ | NA | NA | NA | 1.06 × 10^-3^ |
| **ISV 1998** | 8.9 | 4 | 1.48 × 10^-3^ | 1.66 × 10^-4^ | 6.81 × 10^-4^ | 1.53 × 10^-4^ | NA | NA | 3.58 × 10^-4^ |
| **ISV 1999-01** | 0.2 | 0 | NA | NA | NA | NA | NA | NA | NA |
| **ISV 1999-07** | 0.4 | 0 | NA | NA | NA | NA | NA | NA | NA |
| **ISV 1999-12** | 0.4 | 1 | 3.70 × 10^-4^ | 9.26 × 10^-4^ | 7.58 × 10^-3^ | NA | NA | NA | NA |
| **ISV 2001** | 1.5 | 4 | 1.48 × 10^-3^ | 9.87 × 10^-4^ | 6.06 × 10^-3^ | NA | NA | NA | 2.12 × 10^-3^ |
| **ISV 2008-A** | 6 | 2 | 7.40 × 10^-4^ | 1.23 × 10^-4^ | NA | NA | NA | NA | 1.06 × 10^-3^ |
| **ISV 2008-B** | 6 | 8 | 2.96 × 10^-3^ | 4.94 × 10^-4^ | 2.02 × 10^-3^ | 6.80 × 10^-4^ | NA | 1.34 × 10^-4^ | NA |
| **ISV 2008-B1** | 6 | 6 | 2.22 × 10^-3^ | 3.70 × 10^-4^ | 1.52 × 10^-3^ | 4.54 × 10^-4^ | NA | 1.34 × 10^-4^ | NA |
| **ISV 2008-B1-FTA** | 6 | 6 | 2.22 × 10^-3^ | 3.70 × 10^-4^ | 1.52 × 10^-3^ | 4.54 × 10^-4^ | NA | 1.34 × 10^-4^ | NA |
| **ISV 2008-B2** | 6 | 6 | 2.22 × 10^-3^ | 3.70 × 10^-4^ | 1.52 × 10^-3^ | 4.54 × 10^-4^ | NA | 1.34 × 10^-4^ | NA |
| **ISV 2008-C** | 6 | 6 | 2.22 × 10^-3^ | 3.70 × 10^-4^ | 1.52 × 10^-3^ | 6.80 × 10^-4^ | NA | 1.34 × 10^-4^ | NA |
| **IVV 2008** | 21.4 | 7 | 2.59 × 10^-3^ | 1.21 × 10^-4^ | 2.83 × 10^-4^ | 6.36 × 10^-5^ | 2.72 × 10^-4^ | 7.49 × 10^-5^ | 1.49 × 10^-4^ |
| **IVV 2008-FTA** | 21.4 | 7 | 2.59 × 10^-3^ | 1.21 × 10^-4^ | 2.83 × 10^-4^ | 6.36 × 10^-5^ | 2.72 × 10^-4^ | 7.49 × 10^-5^ | 1.49 × 10^-4^ |
| **CIRAD 2010** | 23 | 7 | 2.59 × 10^-3^ | 1.13 × 10^-4^ | 2.64 × 10^-4^ | 1.18 × 10^-4^ | 2.53 × 10^-4^ | 3.48 × 10^-5^ | 1.38 × 10^-4^ |
