# Supplementary material for: Twenty years of evolution and diversification of digitaria streak virus in Digitaria setigera
Source: Virus Evol. 2021 Oct 13;7(2):veab083. doi: 10.1093/ve/veab083 (PMC8516820; doi:10.1093/ve/veab083)
Supplement: veab083_Supp [file veab083_supp.zip › Supplementary Table S2_Ortega el Campo_VE.docx]

**Supplementary Table S2.** Analysis of genetic variation in DSV mutant spectra.

| **Types of mutation** | **Total samples** | | | |
| --- | --- | --- | --- | --- |
|  | **Obs.^a^** | **Exp.^b^** | **p-value^c^** | **%^d^** |
| **Transversions** | 49 |  |  | 56.32 |
| A → C | 1 | 7.42 | 1.84 × 10^-2^ | 1.15 |
| A → T | 5 | 7.42 | 3.74 × 10^-1^ | 5.75 |
| C → A | 3 | 6.93 | 1.35 × 10^-1^ | 3.45 |
| C → G | 3 | 6.93 | 1.35 × 10^-1^ | 3.45 |
| G → C | 2 | 7.42 | 4.65 × 10^-2^ | 2.30 |
| G → T | 17 | 7.42 | 4.41 × 10^-4^ | 19.54 |
| T → A | 9 | 7.22 | 5.08 × 10^-1^ | 10.34 |
| T → G | 9 | 7.22 | 5.08 × 10^-1^ | 10.34 |
| **Transitions** | 38 |  |  | 43.68 |
| A → G | 6 | 7.42 | 6.01 × 10^-1^ | 6.90 |
| C → T | 18 | 6.93 | 2.62 × 10^-5^ | 20.69 |
| G → A | 9 | 7.42 | 5.63 × 10^-1^ | 10.34 |
| T → C | 5 | 7.22 | 4.09 × 10^-1^ | 5.75 |
| **Total substitutions** | 87 | 87 |  | 87.88 |
| **Insertions^e^** | 6 |  |  | 6.06 |
| **Deletions** | 6 |  |  | 6.06 |
| **Total^f^** | 99 |  |  |  |
| **GC Content** |  |  |  |  |
| G/C→A/T | 47 | 28.71 | 6.41 × 10^-4^ | 54.02 |
| A/T→G/C | 21 | 29.29 | 1.26 × 10^-1^ | 24.14 |
| **Transitions (Ts) vs transversions (Tv)** | | |  |  |
| Ts | 38 | 29.00 | 9.47 × 10^-2^ | 43.68 |
| Tv | 49 | 58.00 | 2.37 × 10^-1^ | 56.32 |
| Ts/Tv | 0.78 |  |  |  |
| **Coding vs non-coding genome regions** | | |  |  |
| Coding | 66 | 80.19 | 1.13 × 10^-1^ | 66.67 |
| Non-coding | 33 | 18.81 | 1.07 × 10^-3^ | 33.33 |

^a^Observed mutations of each type.

^b^Expected mutations of each type.

^c^p-value of the chi^2^ statistical analysis.

^d^Proportion in % of observed mutations.

^e^We considered the mutation INS211-213CCT as a three nt "insertion" since that sequence was deleted in the respective population majority (consensus). Compared to the ancestral DSV sequences (GenBank M23022.1 and MW700085) it represents a 3 nt deletion. See also Figure 2.

^f^For the estimation of the number of mutations and the substitution biases, mutations present in more than one sample have only been counted once, in the chronologically oldest samples.

| **Mutations^a^** | **V2 (MP)** | | **V1 (CP)** | | **C1 (RepA)** | | **C2** | | **LIR** | | **SIR** | | **TOTAL** | |
| --- | --- | --- | --- | --- | --- | --- | --- | --- | --- | --- | --- | --- | --- | --- |
|  | **n** | **%** | **n** | **%** | **n** | **%** | **n** | **%** | **n** | **%** | **n** | **%** | **n** | **%** |
| **Transversions** |  |  |  |  |  |  |  |  |  |  |  |  |  |  |
| Total | 8 | 53.33 | 9 | 39.13 | 13 | 68.42 | 5 | 55.56 | 10 | 76.92 | 4 | 50.00 | 49 | 56.32 |
| A → C | 0 | 0.00 | 1 | 4.35 | 0 | 0.00 | 0 | 0.00 | 0 | 0.00 | 0 | 0.00 | 1 | 1.15 |
| A → T | 0 | 0.00 | 1 | 4.35 | 2 | 10.53 | 0 | 0.00 | 1 | 7.69 | 1 | 12.50 | 5 | 5.75 |
| C → A | 0 | 0.00 | 0 | 0.00 | 2 | 10.53 | 1 | 11.11 | 0 | 0.00 | 0 | 0.00 | 3 | 3.45 |
| C → G | 0 | 0.00 | 1 | 4.35 | 1 | 5.26 | 0 | 0.00 | 1 | 7.69 | 0 | 0.00 | 3 | 3.45 |
| G → C | 1 | 6.67 | 0 | 0.00 | 0 | 0.00 | 0 | 0.00 | 1 | 7.69 | 0 | 0.00 | 2 | 2.30 |
| G → T | 3 | 20.00 | 5 | 21.74 | 4 | 21.05 | 2 | 22.22 | 2 | 15.38 | 1 | 12.50 | 17 | 19.54 |
| T → A | 1 | 6.67 | 1 | 4.35 | 2 | 10.53 | 1 | 11.11 | 3 | 23.08 | 1 | 12.50 | 9 | 10.34 |
| T → G | 3 | 20.00 | 0 | 0.00 | 2 | 10.53 | 1 | 11.11 | 2 | 15.38 | 1 | 12.50 | 9 | 10.34 |
| **Transitions** |  |  |  |  |  |  |  |  |  |  |  |  |  |  |
| Total | 7 | 46.67 | 14 | 60.87 | 6 | 31.58 | 4 | 44.44 | 3 | 23.08 | 4 | 50.00 | 38 | 43.68 |
| A → G | 0 | 0.00 | 2 | 8.70 | 1 | 5.26 | 1 | 11.11 | 1 | 7.69 | 1 | 12.50 | 6 | 6.90 |
| C → T | 5 | 33.33 | 8 | 34.78 | 3 | 15.79 | 1 | 11.11 | 1 | 7.69 | 0 | 0.00 | 18 | 20.69 |
| G → A | 1 | 6.67 | 2 | 8.70 | 1 | 5.26 | 1 | 11.11 | 1 | 7.69 | 3 | 37.50 | 9 | 10.34 |
| T → C | 1 | 6.67 | 2 | 8.70 | 1 | 5.26 | 1 | 11.11 | 0 | 0.00 | 0 | 0.00 | 5 | 5.75 |
| **Ts/Tv** | 0.88 |  | 1.56 |  | 0.46 |  | 0.80 |  | 0.30 |  | 1.00 |  | 0.78 |  |
| **Substitutions** | 15 | 83.33 | 23 | 100.00 | 19 | 100.00 | 9 | 100.00 | 13 | 61.90 | 8 | 88.89 | 87 | 87.88 |
| **Insertions^b^** | 3 | 16.67 | 0 | 0.00 | 0 | 0.00 | 0 | 0.00 | 2 | 9.52 | 1 | 11.11 | 6 | 6.06 |
| **Deletions** | 0 | 0.00 | 0 | 0.00 | 0 | 0.00 | 0 | 0.00 | 6 | 28.57 | 0 | 0.00 | 6 | 6.06 |
| **Total^c^** | 18 |  | 23 |  | 19 |  | 9 |  | 21 |  | 9 |  | 99 |  |
| **Nonsynonymous** | 13 | 72.22 | 8 | 34.78 | 15 | 78.95 | 7 | 77.78 | Na | Na | Na | Na | 43 | 43.43 |
| **Missense** | 12 | 66.67 | 8 | 34.78 | 12 | 63.16 | 7 | 77.78 | Na | Na | Na | Na | 39 | 39.39 |
| **Nonsense** | 1 | 5.56 | 0 | 0.00 | 3 | 15.79 | 0 | 0.00 | Na | Na | Na | Na | 4 | 4.04 |
| **Frameshift** | 0 | 0.00 | 0 | 0.00 | 0 | 0.00 | 0 | 0.00 | Na | Na | Na | Na | 0 | 0.00 |
| **Synonymous** | 2 | 11.11 | 16 | 69.57 | 5 | 26.32 | 3 | 33.33 | Na | Na | Na | Na | 26 | 26.26 |
| **dN/dS** | 4.99 | | 0.17 | | 0.84 | | 1.23 | | Na | | Na | | 0.71 | |
| **Selection pressure** | **Positive** | | **Negative** | | **Negative** | | **Positive** | | Na | | Na | | **Negative** | |

^a^Genetic variability in mutant spectra by genomic regions and in the whole genome.

^b^We considered the mutation INS211-213CCT as a three nt "insertion" since that sequence was deleted in the respective population majority (consensus). Compared to the ancestral DSV sequences (GenBank M23022.1 and MW700085) it represents a 3 nt deletion. See also Figure 2.

^c^For the estimation of the number of mutations in each genomic region, mutations present in more than one sample have only been counted once, in the chronologically oldest samples.
