# Supplementary material for: Twenty years of evolution and diversification of digitaria streak virus in Digitaria setigera
Source: Virus Evol. 2021 Oct 13;7(2):veab083. doi: 10.1093/ve/veab083 (PMC8516820; doi:10.1093/ve/veab083)
Supplement: veab083_Supp [file veab083_supp.zip › Supplementary Table S3_Ortega del Campo et al_VE.docx]

**Supplementary Table S3.** Mutations, codons and amino acid changes present in DSV mutant spectra during the 20 years of evolution in *D. setigera* with respect to their respective consensus sequences.

| **DSV ISV 1990** | | | | | |
| --- | --- | --- | --- | --- | --- |
|  |  |  |  |  |  |
| **DSV 1987_M23022.1 coordinates^a^** | | | **Consensus (quasispecies alignment) coordinates^b^** | | |
| **nt change** | **Codon change** | **Amino acid change** | **nt change** | **Codon change** | **Amino acid change** |
| **V2 (MP)** | | | | | |
| **(nt pos 1 to 330)** | | | **(nt pos 1 to 330)** | | |
| T161A | UUU → UAU | Phe54Tyr | T161A | UUU → UAU | Phe54Tyr |
| T182G | GUA → GGA | Val61Gly | T182G | GUA → GGA | Val61Gly |
| C221T | ACA → AUA | Thr74Ile | C221T | ACA → AUA | Thr74Ile |
| T233C | UUG → UCG | Leu78Ser | T233C | UUG → UCG | Leu78Ser |
| C271T | CCC → UCC | Pro91Ser | C271T | CCC → UCC | Pro91Ser |
| **V1 (CP)** | | | | | |
| **(nt pos 315 to 1049)** | | | **(nt pos 315 to 1049)** | | |
| G486A | GGC → AGC | Gly58Ser | G486A | GGC → AGC | Gly58Ser |
| **C896T^c^** | **CUC → CUU** | **Leu194Leu** | **C896T** | **CUC → CUU** | **Leu194Leu** |
| C959T | GCC → GCU | Ala215Ala | C959T | GCC → GCU | Ala215Ala |
| A1011G | ACC → GCC | Thr233Ala | A1011G | ACC → GCC | Thr233Ala |
| **SIR** | | | | | |
| **(nt pos 1050 to 1222)** | | | **(nt pos 1050 to 1222)** | | |
| G1051A | - | - | G1051A | - | - |
| **C1 (RepA)^d^** | | | | | |
| **(nt pos 1585 to 2385)** | | | **(nt pos 1585 to 2385)** | | |
| T1769G | GAC → GCC | Asp206Ala | T1769G | GAC → GCC | Asp206Ala |
| C1785T | GAU → AAU | Asp201Asn | C1785T | GAU → AAU | Asp201Asn |
| **G1999T^c^** | **CAC → AAC** | **His129Gln** | **G1999T** | **CAC → AAC** | **His129Gln** |
| **LIR** | | | | | |
| **(nt pos 2386 to 2701)** | | | **(nt pos 2386 to 2701)** | | |
| G2388A | - | - | G2388A | - | - |
| T2484G | - | - | T2484G | - | - |
| T2493A | - | - | T2493A | - | - |
| A2494T | - | - | A2494T | - | - |
| T2495A | - | - | T2495A | - | - |
| INS2542G | - | - | INS2542G | - | - |
| INS2615A | - | - | INS2615A | - | - |

^a^The 1987 consensus sequence (M23022.1) was used as coordinates to indicate the position of each mutation.

^b^The consensus sequence obtained from the corresponding mutant spectrum (GenBank Accession Number MW700085, Supplementary Table S8) was used as coordinates to indicate the position of each mutation.

^c^Mutations that appeared in a quasispecies and subsequently fixed in the DSV consensus sequence are shown in bold.

^d^Please note that C1 ORF is on the complementary strand; nucleotides changes (on viral strand) leading to codon changes (on complementary strand) are underlined.

| **DSV ISV 1998** | | | | | |  |
| --- | --- | --- | --- | --- | --- | --- |
|  |  |  |  |  |  |  |
| **DSV 1987_M23022.1 coordinates^a^** | | | **Consensus (quasispecies alignment) coordinates^b^** | | |  |
| **nt change** | **Codon change** | **Amino acid change** | **nt change** | **Codon change** | **Amino acid change** |  |
| **V2 (MP)** | | | | | |  |
| **(nt pos 1 to 330)** | | | **(nt pos 1 to 327)** | | |  |
| G74C | AGU → ACU | Ser25Gly | G74C | AGU → ACU | Ser25Gly |  |
| G141A | UGG → UGA | Trp47Stop | G141A | UGG → UGA | Trp47Stop |  |
| INS211-213CCT | GGU → GG UCC U | GlyINS70Pro | INS211-213CCT | GGU → GG UCC U | GlyINS70Pro |  |
| **TT232-233GG^c^** | **UUG → GGG** | **Leu78Gly** | **TT229-230GG** | **UUG → GGG** | **Leu77Gly** |  |
| **V1 (CP)** | | | | | |  |
| **(nt pos 315 to 1049)** | | | **(nt pos 312 to 1046)** | | |  |
| **A470G^c^** | **ACA → ACG** | **Thr52Thr** | **A467G** | **ACA → ACG** | **Thr52Thr** |  |
| C638T | GUC→ GUU | Val108Val | C635T | GUC→ GUU | Val108Val |  |
| G865A | AGU → AAU | Ser184Asn | G862A | AGU → AAU | Ser184Asn |  |
| C1007T | UUC → UUU | Phe231Phe | C1004T | UUC → UUU | Phe231Phe |  |
| **C2^d, e^** | | | | | |  |
| **(nt pos 1220 to 1666)** | | | **(nt pos 1217 to 1663)** | | |  |
| G1517T | GUC → GUA | Val50Val | G1514T | GUC → GUA | Val50Val |  |
| G1538T | GAC → GAA | Asp43Glu | G1535T | GAC → GAA | Asp43Glu |  |
| C1597T | GCC → ACC | Ala24Thr | C1594T | GCC → ACC | Ala24Thr |  |
| **C1 (RepA)^e^** | | | | | |  |
| **(nt pos 1585 to 2385)** | | | **(nt pos 1582 to 2382)** | | |  |
| C2023A | GAG → GAT | Glu121Asp | C2020A | GAG → GAT | Glu121Asp |  |
| **LIR** | | | | | |  |
| **(nt pos 2386 to 2701)** | | | **(nt pos 2383 to 2698)** | | |  |
| A2494G | - | - | A2491G | - | - |  |
| G2547DEL | - | - | G2544DEL | - | - |  |
| T2691G | - | - | T2688G | - | - |  |
| **C2692G^c^** | **-** | **-** | **C2689G** | **-** | **-** |  |
| G2693T | - | - | G2690T | - | - |  |

^a^The 1987 consensus sequence (M23022.1) was used as coordinates to indicate the position of each mutation.

^b^The consensus sequence obtained from the corresponding mutant spectrum (GenBank Accession Number MW700086, Supplementary Table S8) was used as coordinates to indicate the position of each mutation.

^c^Mutations that appeared in a quasispecies and subsequently fixed in the DSV consensus sequence are shown in bold.

^d^The clustered amino acid changes in the C2 gene correspond to the replication-associated protein (Rep).

^e^Please note that C2 and C1 ORFs are on the complementary strand; nucleotides changes (on viral strand) leading to codon changes (on complementary strand) are underlined.

| **DSV ISV 2001** | | | | | |  |
| --- | --- | --- | --- | --- | --- | --- |
|  |  |  |  |  |  |  |
| **DSV 1987_M23022.1 coordinates^a^** | | | **Consensus (quasispecies alignment) coordinates^b^** | | |  |
| **nt change** | **Codon change** | **Amino acid change** | **nt change** | **Codon change** | **Amino acid change** |  |
| **V2 (MP)** | | | | | |  |
| **(nt pos 1 to 330)** | | | **(nt pos 1 to 327)** | | |  |
| **INS211-213CCT ^d^** | **GGU → GG UCC U** | **GlyINS70Pro** | **INS211-213CCT** | **GGU → GG UCC U** | **GlyINS70Pro** |  |
| GG232-233TT | GGG → UUG | Gly78Leu | GG229-230TT | GGG → UUG | Gly77Leu |  |
| **V1 (CP)** | | | | | |  |
| **(nt pos 315 to 1049)** | | | **(nt pos 312 to 1046)** | | |  |
| T433C | CUC → CCC | Leu40Pro | T430C | CUC → CCC | Leu40Pro |  |
| **A470G^c, d^** | **ACA → ACG** | **Thr52Thr** | **A467G** | **ACA → ACG** | **Thr52Thr** |  |
| A864T | AGU → UGU | Ser184Cys | A861T | AGU → UGU | Ser184Cys |  |
| CG1017-1018TT | CGU → UUU | Arg235Phe | CG1014-1015TT | CGU → UUU | Arg235Phe |  |
| **SIR** | | | | | |  |
| **(nt pos 1050 to 1222)** | | | **(nt pos 1047 to 1219)** | | |  |
| G1063T | - | - | G1060T | - | - |  |
| A1107G | - | - | A1104G | - | - |  |
| INS1199A | - | - | INS1196A | - | - |  |
| **C1 (RepA)^e^** | | | | | |  |
| **(nt pos 1585 to 2385)** | | | **(nt pos 1582 to 2382)** | | |  |
| C1924A | AAG → AAU | Lys154Asn | C1921A | AAG → AAU | Lys154Asn |  |
| G2001T | CAC → AAC | His129Asn | G1998T | CAC → AAC | His129Asn |  |
| T2100C | ACA → GCA | Thr96Ala | T2097C | ACA → GCA | Thr96Ala |  |
| A2248G | GUU → GUC | Val46Val | A2245G | GUU → GUC | Val46Val |  |
| A2272T | UCU → UCA | Ser38Ser | A2269T | UCU → UCA | Ser38Ser |  |
| **LIR** | | | | | |  |
| **(nt pos 2386 to 2701)** | | | **(nt pos 2383 to 2698)** | | |  |
| G2669DEL | - | - | G2666DEL | - | - |  |
| **T2691G^d^** | **-** | **-** | **T2688G** | **-** | **-** |  |
| G2692C | - | - | G2689C | - | - |  |

^a^The 1987 consensus sequence (M23022.1) was used as coordinates to indicate the position of each mutation.

^b^The consensus sequence obtained from the corresponding mutant spectrum (GenBank Accession Number MW700090, Supplementary Table S8) was used as coordinates to indicate the position of each mutation.

^c^Mutations that appeared in a quasispecies and subsequently fixed in the DSV consensus sequence are shown in bold.

^d^Mutations detected in older samples. Since they are samples from the same plant or lineage, we consider that these mutations have been maintained over time since they were first detected. In the analyses indicated in the corresponding tables, we only count the mutation once, when it appeared in the chronologically oldest sample.

^e^Please note that C2 and C1 ORFs are on the complementary strand; nucleotides changes (on viral strand) leading to codon changes (on complementary strand) are underlined.

| **DSV ISV 2008-B1** | | | | | |  |
| --- | --- | --- | --- | --- | --- | --- |
|  |  |  |  |  |  |  |
| **DSV 1987_M23022.1 coordinates^a^** | | | **Consensus (quasispecies alignment) coordinates^b^** | | |  |
| **nt change** | **Codon change** | **Amino acid change** | **nt change** | **Codon change** | **Amino acid change** |  |
| **V1 (CP)** | | | | | |  |
| **(nt pos 315 to 1049)** | | | **(nt pos 312 to 1046)** | | |  |
| G340T | AGG → AGU | Arg9Ser | G337T | AGG → AGU | Arg9Ser |  |
| C587T | CAC → CAU | His91His | C584T | CAC → CAU | His91His |  |
| **C1 (RepA)^c^** | | | | | |  |
| **(nt pos 1585 to 2385)** | | | **(nt pos 1582 to 2382)** | | |  |
| T2072A | AAA → AUA | Lys105Ile | T2069A | AAA → AUA | Lys105Ile |  |
| **LIR** | | | | | |  |
| **(nt pos 2386 to 2701)** | | | **(nt pos 2383 to 2698)** | | |  |
| T2591DEL | - | - | T2588DEL | - | - |  |
| G2668DEL | - | - | G2665DEL | - | - |  |

^a^The 1987 consensus sequence (M23022.1) was used as coordinates to indicate the position of each mutation.

^b^The consensus sequence obtained from the corresponding mutant spectrum (GenBank Accession Number MW700092, Supplementary Table S8) was used as coordinates to indicate the position of each mutation.

^c^Please note that C2 and C1 ORFs are on the complementary strand; nucleotides changes (on viral strand) leading to codon changes (on complementary strand) are underlined.

| **DSV ISV 2008-B1-FTA** | | | | | |  |
| --- | --- | --- | --- | --- | --- | --- |
|  |  |  |  |  |  |  |
| **DSV 1987_M23022.1 coordinates^a^** | | | **Consensus (quasispecies alignment) coordinates^b^** | | |  |
| **nt change** | **Codon change** | **Amino acid change** | **nt change** | **Codon change** | **Amino acid change** |  |
| **V2 (MP)** | | | | | |  |
| **(nt pos 1 to 330)** | | | **(nt pos 1 to 327)** | | |  |
| C67T | CCG → UCG | Pro23Ser | C67T | CCG → UCG | Pro23Ser |  |
| **V1 (CP)** | | | | | |  |
| **(nt pos 315 to 1049)** | | | **(nt pos 312 to 1046)** | | |  |
| C617T | UAC → UAU | Tyr101Tyr | C614T | UAC → UAU | Tyr101Tyr |  |
| **C638T^c^** | **GUC→ GUU** | **Val108Val** | **C635T** | **GUC→ GUU** | **Val108Val** |  |
| T639C | UUG → CUG | Leu109Leu | T636C | UUG → CUG | Leu109Leu |  |
| **SIR** | | | | | |  |
| **(nt pos 1050 to 1222)** | | | **(nt pos 1047 to 1219)** | | |  |
| G1050A | - | - | G1047A | - | - |  |
| **C2^d, e^** | | | | | |  |
| **(nt pos 1220 to 1666)** | | | **(nt pos 1217 to 1663)** | | |  |
| C1287A | AGG → AUG | Arg127Met | C1284A | AGG → AUG | Arg127Met |  |
| **C1 (RepA)^e^** | | | | | |  |
| **(nt pos 1585 to 2385)** | | | **(nt pos 1582 to 2382)** | | |  |
| G1955T | UCA → UAA | Ser144Stop | G1952T | UCA → UAA | Ser144Stop |  |
| **LIR** | | | | | |  |
| **(nt pos 2386 to 2701)** | | | **(nt pos 2383 to 2698)** | | |  |
| C2696T | - | - | C2693T | - | - |  |

^a^The 1987 consensus sequence (M23022.1) was used as coordinates to indicate the position of each mutation.

^b^The consensus sequence obtained from the corresponding mutant spectrum (GenBank Accession Number MW700093, Supplementary Table S8) was used as coordinates to indicate the position of each mutation.

^c^Mutations detected in older samples. Since they are samples from the same plant or lineage, we consider that these mutations have been maintained over time since they were first detected. In the analyses indicated in the corresponding tables, we only count the mutation once, when it appeared in the chronologically oldest sample.

^d^The clustered amino acid changes in the C2 gene correspond to the replication-associated protein (Rep).

^e^Please note that C2 and C1 ORFs are on the complementary strand; nucleotides changes (on viral strand) leading to codon changes (on complementary strand) are underlined.

| **DSV ISV 2008-B2** | | | | | |  |
| --- | --- | --- | --- | --- | --- | --- |
|  |  |  |  |  |  |  |
| **DSV 1987_M23022.1 coordinates^a^** | | | **Consensus (quasispecies alignment) coordinates^b^** | | |  |
| **nt change** | **Codon change** | **Amino acid change** | **nt change** | **Codon change** | **Amino acid change** |  |
| **V2 (MP)** | | | | | |  |
| **(nt pos 1 to 330)** | | | **(nt pos 1 to 327)** | | |  |
| G273T | CCG → CCU | Pro91Pro | G270T | CCG → CCU | Pro90Pro |  |
| **V1 (CP)** | | | | | |  |
| **(nt pos 315 to 1049)** | | | **(nt pos 312 to 1046)** | | |  |
| C368G | CGC → CGG | Arg18Arg | C365G | CGC → CGG | Arg18Arg |  |
| G650T | GUG → GUU | Val112Val | G647T | GUG → GUU | Val112Val |  |
| **SIR** | | | | | |  |
| **(nt pos 1050 to 1222)** | | | **(nt pos 1047 to 1219)** | | |  |
| A1137T | - | - | A1134T | - | - |  |
| **INS1199A^c^** | **-** | **-** | **INS1196A** | **-** | **-** |  |
| **C2^d, e^** | | | | | |  |
| **(nt pos 1220 to 1666)** | | | **(nt pos 1217 to 1663)** | | |  |
| T1370C | GAA → GAG | Glu99Glu | T1367C | GAA → GAG | Glu99Glu |  |
| A1430G | GGU → GGC | Gly79Gly | A1427G | GGU → GGC | Gly79Gly |  |
| T1564G | AAU → CAU | Asn35His | T1561G | AAU → CAU | Asn35His |  |
| **C1 (RepA)^e^** | | | | | |  |
| **(nt pos 1585 to 2385)** | | | **(nt pos 1582 to 2382)** | | |  |
| C1593G | AGA → ACA | Arg25Thr | C1590G | AGA → ACA | Arg25Thr |  |
| C1685T | UGG → UAG | Trp234Stop | C1682T | UGG→UAG | Trp234Stop |  |
| **C1785T^c^** | **GAU → AAU** | **Asp201Asn** | **C1782T** | **GAU→ AAU** | **Asp201Asn** |  |
| T1871A | GAU → GUU | Asp172Val | T1868A | GAU → GUU | Asp172Val |  |
| T1999G | CAA → CAC | Gln129His | T1996G | CAA → CAC | Gln129His |  |
| G2281T | CUC → CUA | Leu35Leu | G2278T | CUC → CUA | Leu35Leu |  |
| **LIR** | | | | | |  |
| **(nt pos 2386 to 2701)** | | | **(nt pos 2383 to 2698)** | | |  |
| T2508A | - | - | T2505A | - | - |  |

^a^The 1987 consensus sequence (M23022.1) was used as coordinates to indicate the position of each mutation.

^b^The consensus sequence obtained from the corresponding mutant spectrum (GenBank Accession Number MW700091, Supplementary Table S8) was used as coordinates to indicate the position of each mutation.

^c^Mutations detected in older samples. Since they are samples from the same plant or lineage, we consider that these mutations have been maintained over time since they were first detected. In the analyses indicated in the corresponding tables, we only count the mutation once, when it appeared in the chronologically oldest sample.

^d^The clustered amino acid changes in the C2 gene correspond to the replication-associated protein (Rep).

^e^Please note that C2 and C1 ORFs are on the complementary strand; nucleotides changes (on viral strand) leading to codon changes (on complementary strand) are underlined.

| **DSV IVV 2008** | | | | | |  |
| --- | --- | --- | --- | --- | --- | --- |
|  |  |  |  |  |  |  |
| **DSV 1987_M23022.1 coordinates^a^** | | | **Consensus (quasispecies alignment) coordinates^b^** | | |  |
| **nt change** | **Codon change** | **Amino acid change** | **nt change** | **Codon change** | **Amino acid change** |  |
| **V2 (MP)** | | | | | |  |
| **(nt pos 1 to 330)** | | | **(nt pos 1 to 330)** | | |  |
| C254T | CCU → CUU | Pro85Leu | C254T | CCU → CUU | Pro85Leu |  |
| **V1 (CP)** | | | | | |  |
| **(nt pos 315 to 1049)** | | | **(nt pos 315 to 1049)** | | |  |
| G743T | GUG → GUU | Val143Val | G743T | GUG → GUU | Val143Val |  |
| **SIR** | | | | | |  |
| **(nt pos 1050 to 1222)** | | | **(nt pos 1050 to 1222)** | | |  |
| T1050G | - | - | T1050G | - | - |  |
| G1087A | - | - | G1087A | - | - |  |
| T1197A | - | - | T1197A | - | - |  |
| **C2^d, e^** | | | | | |  |
| **(nt pos 1220 to 1666)** | | | **(nt pos 1220 to 1666)** | | |  |
| G1249A | CUC → UUC | Leu140Phe | G1249A | CUC → UUC | Leu140Phe |  |
| **C1 (RepA)^e^** | | | | | |  |
| **(nt pos 1585 to 2385)** | | | **(nt pos 1585 to 2385)** | | |  |
| G1850A | GCU → GUU | Ala179Val | G1850A | GCU → GUU | Ala179Val |  |
| **C2023A^c^** | **GAG → GAU** | **Glu121Asp** | **C2023A** | **GAG → GAU** | **Glu121Asp** |  |
| A2035T | CCU → CCA | Pro117Pro | A2035T | CCU → CCA | Pro117Pro |  |

^a^The 1987 consensus sequence (M23022.1) was used as coordinates to indicate the position of each mutation.

^b^The consensus sequence obtained from the corresponding mutant spectrum (GenBank Accession Number MW700097, Supplementary Table S8) was used as coordinates to indicate the position of each mutation.

^c^Mutations detected in older samples. Since they are samples from the same plant or lineage, we consider that these mutations have been maintained over time since they were first detected. In the analyses indicated in the corresponding tables, we only count the mutation once, when it appeared in the chronologically oldest sample.

^d^The clustered amino acid changes in the C2 gene correspond to the replication-associated protein (Rep).

^e^Please note that C2 and C1 ORFs are on the complementary strand; nucleotides changes (on viral strand) leading to codon changes (on complementary strand) are underlined.

| **DSV CIRAD 2010** | | | | | |  |
| --- | --- | --- | --- | --- | --- | --- |
|  |  |  |  |  |  |  |
| **DSV 1987_M23022.1 coordinates^a^** | | | **Consensus (quasispecies alignment) coordinates^b^** | | |  |
| **nt change** | **Codon change** | **Amino acid change** | **nt change** | **Codon change** | **Amino acid change** |  |
| **V2 (MP)** | | | | | |  |
| **(nt pos 1 to 330)** | | | **(nt pos 1 to 330)** | | |  |
| C103T | CUA → UUA | Leu35Leu | C103T | CUA → UUA | Leu35Leu |  |
| **V1 (CP)** | | | | | |  |
| **(nt pos 315 to 1049)** | | | **(nt pos 315 to 1049)** | | |  |
| T602A | GCU → GCA | Ala96Ala | T602A | GCU → GCA | Ala96Ala |  |
| G636T | GUC → UUC | Val108Phe | G636T | GUC → UUC | Val108Phe |  |
| A659C | GCA → GCC | Ala115Ala | A659C | GCA → GCC | Ala115Ala |  |
| C714T | CUA → UUA | Leu134Leu | C714T | CUA → UUA | Leu134Leu |  |
| **C2^d, e^** | | | | | |  |
| **(nt pos 1220 to 1666)** | | | **(nt pos 1221 to 1667)** | | |  |
| C1266T | GGG → GAG | Gly134Glu | C1267T | GGG → GAG | Gly134Glu |  |
| T1279A | AGC → UGC | Ser130Cys | T1280A | AGC → UGC | Ser130Cys |  |
| **LIR** | | | | | |  |
| **(nt pos 2386 to 2701)** | | | **(nt pos 2387 to 2702)** | | |  |
| G2478T | - | - | G2479T | - | - |  |
| C2591DEL | - | - | C2592DEL | - | - |  |
| A2619DEL | - | - | A2620DEL | - | - |  |
| **G2669DEL^c^** | **-** | **-** | **G2670DEL** | **-** | **-** |  |

^a^The 1987 consensus sequence (M23022.1) was used as coordinates to indicate the position of each mutation.

^b^The consensus sequence obtained from the corresponding mutant spectrum (GenBank Accession Number MW700099, Supplementary Table S8) was used as coordinates to indicate the position of each mutation.

^c^Mutations detected in older samples. Since they are samples from the same plant or lineage, we consider that these mutations have been maintained over time since they were first detected. In the analyses indicated in the corresponding tables, we only count the mutation once, when it appeared in the chronologically oldest sample.

^d^The clustered amino acid changes in the C2 gene correspond to the replication-associated protein (Rep).

^e^Please note that C2 and C1 ORFs are on the complementary strand; nucleotides changes (on viral strand) leading to codon changes (on complementary strand) are underlined.
