# Supplementary material for: Twenty years of evolution and diversification of digitaria streak virus in Digitaria setigera
Source: Virus Evol. 2021 Oct 13;7(2):veab083. doi: 10.1093/ve/veab083 (PMC8516820; doi:10.1093/ve/veab083)
Supplement: veab083_Supp [file veab083_supp.zip › Supplementary Table S4_Ortega del Campo_VE.docx]

**Supplementary Table S4.** Genetic complexity and haplotype heterogeneity obtained for the sequenced mutant spectra for the different genomic regions.

| **V2 (MP)** | **Mutation frequency (mut/nt)^a^** |  |  | **C1 (RepA)** | **Mutation frequency (mut/nt)^a^** |
| --- | --- | --- | --- | --- | --- |
| **ISV 1990** | 7.58 × 10^-4^ |  |  | **ISV 1990** | 1.94 × 10^-4^ |
| **ISV 1998** | 1.12 × 10^-3^ |  |  | **ISV 1998** | 7.27 × 10^-5^ |
| **ISV 2001** | 2.77 × 10^-4^ |  |  | **ISV 2001** | 2.85 × 10^-4^ |
| **ISV 2008-B1** | NA |  |  | **ISV 2008-B1** | 5.67 × 10^-5^ |
| **ISV 2008-B1-FTA** | 1.61 × 10^-4^ |  |  | **ISV 2008-B1-FTA** | 6.78 × 10^-5^ |
| **ISV 2008-B2** | 1.53 × 10^-4^ |  |  | **ISV 2008-B2** | 3.49 × 10^-4^ |
| **IVV 2008** | 1.68 × 10^-4^ |  |  | **IVV 2008** | 1.48 × 10^-4^ |
| **CIRAD 2010** | 1.38 × 10^-4^ |  |  | **CIRAD 2010** | NA |
|  |  |  |  |  |  |
| Median | **3.07** **× 10^-4^** |  |  | Median | **1.47 × 10^-4^** |
|  |  |  |  |  |  |
|  |  |  |  |  |  |
|  |  |  |  |  |  |
| **V1 (CP)** | **Mutation frequency (mut/nt)^a^** |  |  | **C2** | **Mutation frequency (mut/nt)^a^** |
| **ISV 1990** | 2.98 × 10^-4^ |  |  | **ISV 1990** | NA |
| **ISV 1998** | 3.41 × 10^-4^ |  |  | **ISV 1998** | 4.73 × 10^-4^ |
| **ISV 2001** | 3.35 × 10^-4^ |  |  | **ISV 2001** | NA |
| **ISV 2008-B1** | 1.55 × 10^-4^ |  |  | **ISV 2008-B1** | NA |
| **ISV 2008-B1-FTA** | 1.89 × 10^-4^ |  |  | **ISV 2008-B1-FTA** | 1.19 × 10^-4^ |
| **ISV 2008-B2** | 1.66 × 10^-4^ |  |  | **ISV 2008-B2** | 3.80 × 10^-4^ |
| **IVV 2008** | 7.79 × 10^-5^ |  |  | **IVV 2008** | 1.28 × 10^-4^ |
| **CIRAD 2010** | 2.66 × 10^-4^ |  |  | **CIRAD 2010** | 2.03 × 10^-4^ |
|  |  |  |  |  |  |
| Median | **2.28 × 10^-4^** |  |  | Median | **1.63 × 10^-4^** |
|  |  |  |  |  |  |
|  |  |  |  |  |  |
|  |  |  |  |  |  |
| **SIR** | **Mutation frequency (mut/nt)^a^** |  |  | **LIR** | **Mutation frequency (mut/nt)^a^** |
| **ISV 1990** | 2.89 × 10^-4^ |  |  | **ISV 1990** | 1.22 × 10^-3^ |
| **ISV 1998** | NA |  |  | **ISV 1998** | 8.93 × 10^-4^ |
| **ISV 2001** | 8.26 × 10^-4^ |  |  | **ISV 2001** | 3.31 × 10^-4^ |
| **ISV 2008-B1** | NA |  |  | **ISV 2008-B1** | 3.24 × 10^-4^ |
| **ISV 2008-B1-FTA** | 3.04 × 10^-4^ |  |  | **ISV 2008-B1-FTA** | 1.80 × 10^-4^ |
| **ISV 2008-B2** | 3.21 × 10^-4^ |  |  | **ISV 2008-B2** | 1.69 × 10^-4^ |
| **IVV 2008** | 9.63 × 10^-4^ |  |  | **IVV 2008** | NA |
| **CIRAD 2010** | NA |  |  | **CIRAD 2010** | 4.62 × 10^-4^ |
|  |  |  |  |  |  |
| Median | **3.38** **× 10^-4^** |  |  | Median | **4.47** **× 10^-4^** |

**^a^**To estimate mutation frequency on each genomic region, mutations present in more than one sample have only been counted once, in the chronologically oldest samples.

| **V2 (MP)** | **Genetic distance** | |  | **C1 (RepA)** | **Genetic distance** | |
| --- | --- | --- | --- | --- | --- | --- |
|  | **d** | **s.e.** |  |  | **d** | **s.e.** |
| **ISV 1990** | 0.00348 | 0.00346 |  | **ISV 1990** | 0.00036 | 0.00081 |
| **ISV 1998** | 0.00316 | 0.00344 |  | **ISV 1998** | 0.00024 | 0.00057 |
| **ISV 2001** | 0.00310 | 0.00308 |  | **ISV 2001** | 0.00177 | 0.00283 |
| **ISV 2008-B1** | 0.00000 | 0.00000 |  | **ISV 2008-B1** | 0.00011 | 0.00036 |
| **ISV 2008-B1-FTA** | 0.00032 | 0.00094 |  | **ISV 2008-B1-FTA** | 0.00012 | 0.00038 |
| **ISV 2008-B2** | 0.00058 | 0.00120 |  | **ISV 2008-B2** | 0.00094 | 0.00114 |
| **IVV 2008** | 0.00034 | 0.00096 |  | **IVV 2008** | 0.00114 | 0.00120 |
| **CIRAD 2010** | 0.00075 | 0.00131 |  | **CIRAD 2010** | 0.00000 | 0.00000 |
|  |  |  |  |  |  |  |
| Median | **0.00147** | **0.00180** |  | Median | **0.00059** | **0.00091** |
|  |  |  |  |  |  |  |
|  |  |  |  |  |  |  |
|  |  |  |  |  |  |  |
| **V1 (CP)** | **Genetic distance** | |  | **C2** | **Genetic distance** | |
|  | **d** | **s.e.** |  |  | **d** | **s.e.** |
| **ISV 1990** | 0.00084 | 0.00096 |  | **ISV 1990** | 0.00000 | 0.00000 |
| **ISV 1998** | 0.00091 | 0.00115 |  | **ISV 1998** | 0.00240 | 0.00943 |
| **ISV 2001** | 0.00178 | 0.00165 |  | **ISV 2001** | 0.00000 | 0.00000 |
| **ISV 2008-B1** | 0.00049 | 0.00117 |  | **ISV 2008-B1** | 0.00000 | 0.00000 |
| **ISV 2008-B1-FTA** | 0.00059 | 0.00104 |  | **ISV 2008-B1-FTA** | 0.00024 | 0.00070 |
| **ISV 2008-B2** | 0.00028 | 0.00069 |  | **ISV 2008-B2** | 0.00161 | 0.00219 |
| **IVV 2008** | 0.00012 | 0.00040 |  | **IVV 2008** | 0.00024 | 0.00072 |
| **CIRAD 2010** | 0.00083 | 0.00099 |  | **CIRAD 2010** | 0.00059 | 0.00103 |
|  |  |  |  |  |  |  |
| Median | **0.00073** | **0.00101** |  | Median | **0.00063** | **0.00176** |
|  |  |  |  |  |  |  |
|  |  |  |  |  |  |  |
|  |  |  |  |  |  |  |
| **SIR** | **Genetic distance** | |  | **LIR** | **Genetic distance** | |
|  | **d** | **s.e.** |  |  | **d** | **s.e.** |
| **ISV 1990** | 0.00058 | 0.00175 |  | **ISV 1990** | 0.00559 | 0.00538 |
| **ISV 1998** | 0.00000 | 0.00000 |  | **ISV 1998** | 0.00315 | 0.00316 |
| **ISV 2001** | 0.00177 | 0.00283 |  | **ISV 2001** | 0.00339 | 0.00354 |
| **ISV 2008-B1** | 0.00000 | 0.00000 |  | **ISV 2008-B1** | 0.00000 | 0.00000 |
| **ISV 2008-B1-FTA** | 0.00061 | 0.00179 |  | **ISV 2008-B1-FTA** | 0.00038 | 0.00111 |
| **ISV 2008-B2** | 0.00065 | 0.00183 |  | **ISV 2008-B2** | 0.00034 | 0.00104 |
| **IVV 2008** | 0.00510 | 0.00524 |  | **IVV 2008** | 0.00000 | 0.00000 |
| **CIRAD 2010** | 0.00000 | 0.00000 |  | **CIRAD 2010** | 0.00031 | 0.00100 |
|  |  |  |  |  |  |  |
| Median | **0.00109** | **0.00168** |  | Median | **0.00165** | **0.00190** |

| **V2 (MP)** | **Shannon index** |  |  | **C1 (RepA)** | **Shannon index** |
| --- | --- | --- | --- | --- | --- |
| **ISV 1990** | 0.41 |  |  | **ISV 1990** | 0.13 |
| **ISV 1998** | 0.43 |  |  | **ISV 1998** | 0.07 |
| **ISV 2001** | 0.27 |  |  | **ISV 2001** | 0.29 |
| **ISV 2008-B1** | 0.00 |  |  | **ISV 2008-B1** | 0.06 |
| **ISV 2008-B1-FTA** | 0.07 |  |  | **ISV 2008-B1-FTA** | 0.07 |
| **ISV 2008-B2** | 0.11 |  |  | **ISV 2008-B2** | 0.39 |
| **IVV 2008** | 0.07 |  |  | **IVV 2008** | 0.35 |
| **CIRAD 2010** | 0.13 |  |  | **CIRAD 2010** | 0.00 |
|  |  |  |  |  |  |
| Median | **0.19** |  |  | Median | **0.17** |
|  |  |  |  |  |  |
|  |  |  |  |  |  |
|  |  |  |  |  |  |
| **V1 (CP)** | **Shannon index** |  |  | **C2** | **Shannon index** |
| **ISV 1990** | 0.33 |  |  | **ISV 1990** | 0.00 |
| **ISV 1998** | 0.39 |  |  | **ISV 1998** | 0.22 |
| **ISV 2001** | 0.37 |  |  | **ISV 2001** | 0.00 |
| **ISV 2008-B1** | 0.11 |  |  | **ISV 2008-B1** | 0.00 |
| **ISV 2008-B1-FTA** | 0.24 |  |  | **ISV 2008-B1-FTA** | 0.07 |
| **ISV 2008-B2** | 0.13 |  |  | **ISV 2008-B2** | 0.27 |
| **IVV 2008** | 0.07 |  |  | **IVV 2008** | 0.07 |
| **CIRAD 2010** | 0.41 |  |  | **CIRAD 2010** | 0.16 |
|  |  |  |  |  |  |
| Median | **0.26** |  |  | Median | **0.10** |
|  |  |  |  |  |  |
|  |  |  |  |  |  |
|  |  |  |  |  |  |
| **SIR** | **Shannon index** |  |  | **LIR** | **Shannon index** |
| **ISV 1990** | 0.07 |  |  | **ISV 1990** | 0.61 |
| **ISV 1998** | 0.00 |  |  | **ISV 1998** | 0.57 |
| **ISV 2001** | 0.25 |  |  | **ISV 2001** | 0.39 |
| **ISV 2008-B1** | 0.00 |  |  | **ISV 2008-B1** | 0.19 |
| **ISV 2008-B1-FTA** | 0.07 |  |  | **ISV 2008-B1-FTA** | 0.07 |
| **ISV 2008-B2** | 0.17 |  |  | **ISV 2008-B2** | 0.07 |
| **IVV 2008** | 0.38 |  |  | **IVV 2008** | 0.00 |
| **CIRAD 2010** | 0.00 |  |  | **CIRAD 2010** | 0.38 |
|  |  |  |  |  |  |
| Median | **0.12** |  |  | Median | **0.28** |
