# Supplementary material for: Twenty years of evolution and diversification of digitaria streak virus in Digitaria setigera
Source: Virus Evol. 2021 Oct 13;7(2):veab083. doi: 10.1093/ve/veab083 (PMC8516820; doi:10.1093/ve/veab083)
Supplement: veab083_Supp [file veab083_supp.zip › Supplementary Table S5_Ortega del Campo_VE.docx]

**Supplementary Table S5.** Analysis of synonymous and non-synonymous mutations (dN/dS) of the open reading frames of consensus sequences and all sequenced mutant spectra.

|  | **Mutations in consensus sequences^a^** | | | | | |
| --- | --- | --- | --- | --- | --- | --- |
|  | **Genomic region** | | | | | **Total** |
|  | **V2 (MP)** | **V1 (CP)** | **C1 (RepA)** | **C2** | **C1/C2** |  |
| **Synonymous (Sd) mutations** | 1 | 7 | 1 | 1 | 2 | 10 |
| **Non-synonymous (Nd) mutations** | 11 | 0 | 2 | 0 | 2 | 13 |
| **S** | 10.330 | 6.330 | 1.670 | 1.000 | 2.670 | 19.33 |
| **NS** | 25.670 | 14.670 | 7.330 | 2.000 | 9.330 | 49.67 |
| **pS** | 0.097 | 1.106 | 0.599 | 1.000 | 0.749 | 0.517 |
| **pN** | 0.429 | 0.000 | 0.273 | 0.000 | 0.214 | 0.262 |
| **dS** | 0.104 |  | 1.201 |  | 5.014 | 0.878 |
| **dN** | 0.635 | 0.000 | 0.339 | 0.000 | 0.252 | 0.322 |
| **dN/dS** | 6.130 |  | 0.282 |  | 0.050 | 0.367 |
| **Selection pressure** | **Positive selection** |  | **Negative selection** |  | **Negative selection** | **Negative selection** |

^a^Mutations that were fixed in separate consensus sequences were counted only once.

Sd: number of observed synonymous mutations

Nd: number of observed non-synonymous mutations

S: total number of synonymous sites

NS: total number of non-synonymous sites

pS: proportion of synonymous differences

pN: proportion of non-synonymous differences

dS: number of synonymous mutations per synonymous site

dN: number of non-synonymous mutations per non-synonymous site

dN/dS: ratio of non-synonymous to synonymous mutations

|  | **Mutations in mutant spectra^a, b^** | | | | | |
| --- | --- | --- | --- | --- | --- | --- |
|  | **Genomic region** | | | | | **Total** |
|  | **V2 (MP)** | **V1 (CP)** | **C1 (RepA)** | **C2** | **C1/C2** |  |
| **Synonymous (Sd) mutations** | 2 | 16 | 5 | 3 | 8 | 26 |
| **Non-synonymous (Nd) mutations** | 13 | 8 | 15 | 7 | 22 | 43 |
| **S** | 15.999 | 26.664 | 13.663 | 10.332 | 23.995 | 66.658 |
| **NS** | 29.001 | 45.336 | 46.337 | 20.668 | 67.005 | 141.342 |
| **pS** | 0.125 | 0.600 | 0.366 | 0.290 | 0.333 | 0.390 |
| **pN** | 0.448 | 0.176 | 0.324 | 0.339 | 0.328 | 0.304 |
| **dS** | 0.137 | 1.207 | 0.502 | 0.367 | 0.441 | 0.551 |
| **dN** | 0.683 | 0.201 | 0.424 | 0.451 | 0.432 | 0.390 |
| **dN/dS** | 4.994 | 0.167 | 0.844 | 1.227 | 0.979 | 0.709 |
| **Selection pressure** | **Positive selection** | **Negative selection** | **Negative selection** | **Positive selection** | **Neutral selection** | **Negative selection** |

^a^Mutations were counted only once in each mutant spectrum.

^b^All mutations were considered for this analysis, including repeats.

Sd: number of observed synonymous mutations

Nd: number of observed non-synonymous mutations

S: total number of synonymous sites

NS: total number of non-synonymous sites

pS: proportion of synonymous differences

pN: proportion of non-synonymous differences

dS: number of synonymous mutations per synonymous site

dN: number of non-synonymous mutations per non-synonymous site

dN/dS: ratio of non-synonymous to synonymous mutations
