# Supplementary material for: Twenty years of evolution and diversification of digitaria streak virus in Digitaria setigera
Source: Virus Evol. 2021 Oct 13;7(2):veab083. doi: 10.1093/ve/veab083 (PMC8516820; doi:10.1093/ve/veab083)
Supplement: veab083_Supp [file veab083_supp.zip › Supplementary Table S6_Ortega del Campo_VE.docx]

**Supplementary Table S6.** Diversifying and purifying selection on DSV. Analysis of selection pressure of the DSV genomic regions using SLAC, FEL, MEME (p-value = 0.2) and FUBAR (posterior probability = 0.9) methods.

| **Gene ORF** | **Codon position** | **SLAC^a^** | | | **FEL** | | **FUBAR^b^** | | | **MEME^c^** | | **Inferred substitution** |  |
| --- | --- | --- | --- | --- | --- | --- | --- | --- | --- | --- | --- | --- | --- |
|  |  |  |  |  |  |  |  |  |  |  |  |  |  |
|  |  | **dN-dS** | **p[dN/dS < 1]** | **p[dN/dS > 1]** | **dN/dS** | **p-value** | **dN-dS** | **Prob[dS < dN]** | **Prob[dS > dN]** | **dN/neutral evolution component** | **p-value** |  |  |
|  |  |  |  |  |  |  |  |  |  |  |  |  |  |
| *MP (V2)* | *78* | *22.9* | *Na* | *0.48* | *infinity* | *0.2* | *16.5* | *0.9* | *Na* | *98.65* | *0.1* | *Ser -> Leu/Gly -> Leu* |  |
| MP (V2) | 35 | -10.5 | 0.5 | Na | 0 | 0.14 | -12 | Na | 0.86 | Na | Na | CTA -> TTA (Leu) |  |
| MP (V2) | 71 | -23.2 | 0.33 | Na | 0 | 0.15 | -12 | Na | 0.86 | Na | Na | CCC -> CCT (Pro) |  |
|  |  |  |  |  |  |  |  |  |  |  |  |  |  |
| *CP (V1)* | *184* | *19.1* | *Na* | *0.6* | *infinity* | *0.2* | *17.8* | *0.95* | *Na* | *9.33* | *0.2* | *Ser -> Asn* |  |
| *CP (V1)* | *235* | *22.2* | *Na* | *0.42* | *infinity* | *0.11* | *15.5* | *0.95* | *Na* | *64.57* | *0.04* | *Arg -> Phe* |  |
| CP (V1) | 112 | -27.9 | 0.33 | Na | 0 | 0.09 | -18.2 | Na | 0.92 | Na | Na | GTG -> GTT (Val) |  |
| CP (V1) | **115** | **-55.7** | **0.1** | **Na** | **0** | **0.02** | **-33.1** | **Na** | **0.99** | **Na** | **Na** | **GCA -> GCC (Val)** |  |
| CP (V1) | 143 | -25.7 | 0.33 | Na | 0 | 0.09 | -17.5 | Na | 0.91 | Na | Na | GTG -> GTT (Val) |  |
| CP (V1) | **194** | **-44.8** | **0.33** | **Na** | **0** | **0.035** | **-20.4** | **Na** | **0.98** | **Na** | **Na** | **CTC -> CTT (Leu)** |  |
|  |  |  |  |  |  |  |  |  |  |  |  |  |  |
| RepA (C1) | 35 | -35.1 | 0.34 | Na | 0 | 0.11 | -22.1 | Na | 0.92 | Na | Na | CTC -> CTA (Leu) |  |
| RepA (C1) | 38 | -35.5 | 0.34 | Na | 0 | 0.13 | -20.3 | Na | 0.91 | Na | Na | TCT -> TCA (Ser) |  |
| RepA (C1) | 46 | -35.5 | 0.33 | Na | 0 | 0.12 | -20.6 | Na | 0.91 | Na | Na | GTT -> GTC (Val) |  |
| RepA (C1) | 83 | -35.5 | 0.33 | Na | 0 | 0.22 | -16 | Na | 0.88 | Na | Na | CCA -> CCT (Pro) |  |
| RepA (C1) | **117** | **-71.1** | **0.11** | **Na** | **0** | **0.08** | **-31.4** | **Na** | **0.98** | **Na** | **Na** | **CCA -> CCT (Pro)** |  |
| RepA (C1) | 263 | -39.7 | 0.33 | Na | 0 | 0.12 | -21.4 | Na | 0.91 | Na | Na | GGG -> GGA (Gly) |  |
|  |  |  |  |  |  |  |  |  |  |  |  |  |  |
| (C2)^d^ | 50 | -32.2 | 0.33 | Na | 0 | 0.14 | -20.2 | Na | 0.9 | Na | Na | GTC -> GTA (Leu) |  |
| (C2) | **79** | **-64.3** | **0.11** | **Na** | **0** | **0.045** | **-34.1** | **Na** | **0.98** | **Na** | **Na** | **GGT -> GGC (Gly)** |  |
| (C2) | **99** | **-93.6** | **0.12** | **Na** | **0** | **0.042** | **-26.2** | **Na** | **0.94** | **Na** | **Na** | **GAA -> GAG (Glu)** |  |
| (C2) | **132** | **-64.2** | **0.11** | **Na** | **0** | **0.049** | **-34** | **Na** | **0.98** | **Na** | **Na** | **GCA -> GCT (Ala)** |  |

^a^In SLAC (DataMonkey server), the statistical test for codon sites under negative or purifying selection was estimated from a p[dN/dS < 1] value, while the statistical test for codon sites under positive or diversifying selection pressure was estimated from a p[dN/dS > 1] value.

^b^In FUBAR (DataMonkey server), the statistical test for codon sites under negative or purifying selection was estimated from a Prob[dS > dN] value, while the statistical test for codon sites under positive or diversifying selection pressure was estimated from a Prob[dS < dN].

^c^MEME aims to detect only sites evolving under positive selection.

^d^ORF C2 is expressed by differential splicing from ORFs C1 and C2 yielding the protein Rep. ORF C2 is not individually expressed into protein. Protein Rep A is expressed from a non-spliced mRNA (Brown et al. 2012). For this analysis, the complete ORFs were used, not taking into account any splicing.

The three positions showing support for diversifying (positive) selection are indicated by italics and blue background.

Positions marked in bold show strong negative selection.
