# Supplementary material for: Twenty years of evolution and diversification of digitaria streak virus in Digitaria setigera
Source: Virus Evol. 2021 Oct 13;7(2):veab083. doi: 10.1093/ve/veab083 (PMC8516820; doi:10.1093/ve/veab083)
Supplement: veab083_Supp [file veab083_supp.zip › Supplementary Table S7_Ortega del Campo_VE.docx]

**Supplementary Table S7.** Recombination analysis of DSV mutant spectra.

| **Recombinant virus** | **Recombination**  **Break points** | **Gene/Region^1^** | **Parent sequences** | | **Methods that detected**  **recombination^2^** | **Lowest p-value** |
| --- | --- | --- | --- | --- | --- | --- |
|  |  |  | **Major** | **Minor** |  |  |
| **ISV 1990 Clone 10^3^** | **691, 1852** | **CP, C1 (RepA)** | **ISV 1990 Clone 14** | **ISV 1990 Clone 17** | **G, B, M, 3S** | **7.39 x 10^-5^** |
| **ISV 1998 Clone 16^4^** | **1065, 2697** | **SIR, LIR** | **ISV 1998 Clone 28** | **ISV 1998 Clone 23** | **G, B, M, 3S** | **4.71 x 10^-5^** |

^1^Genomic regions where the recombination break points mapped

^2^R (RDP); G (GENCONV); B (BootScan); M (MaxChi); C (Chimaera); S (SiScan) and 3S (3Seq)

^3^Recombinant virus that in addition contained a sequence in SIR (positions 1111 to 1207) from a DSV sequence from the ORFs C1/C2 (positions 1686 to 1590)

^4^Recombinant virus that in addition contained a sequence in ORF C1 (positions 1896 to 2180) from a DSV sequence from the ORF C1 (positions 1590 to 1844)


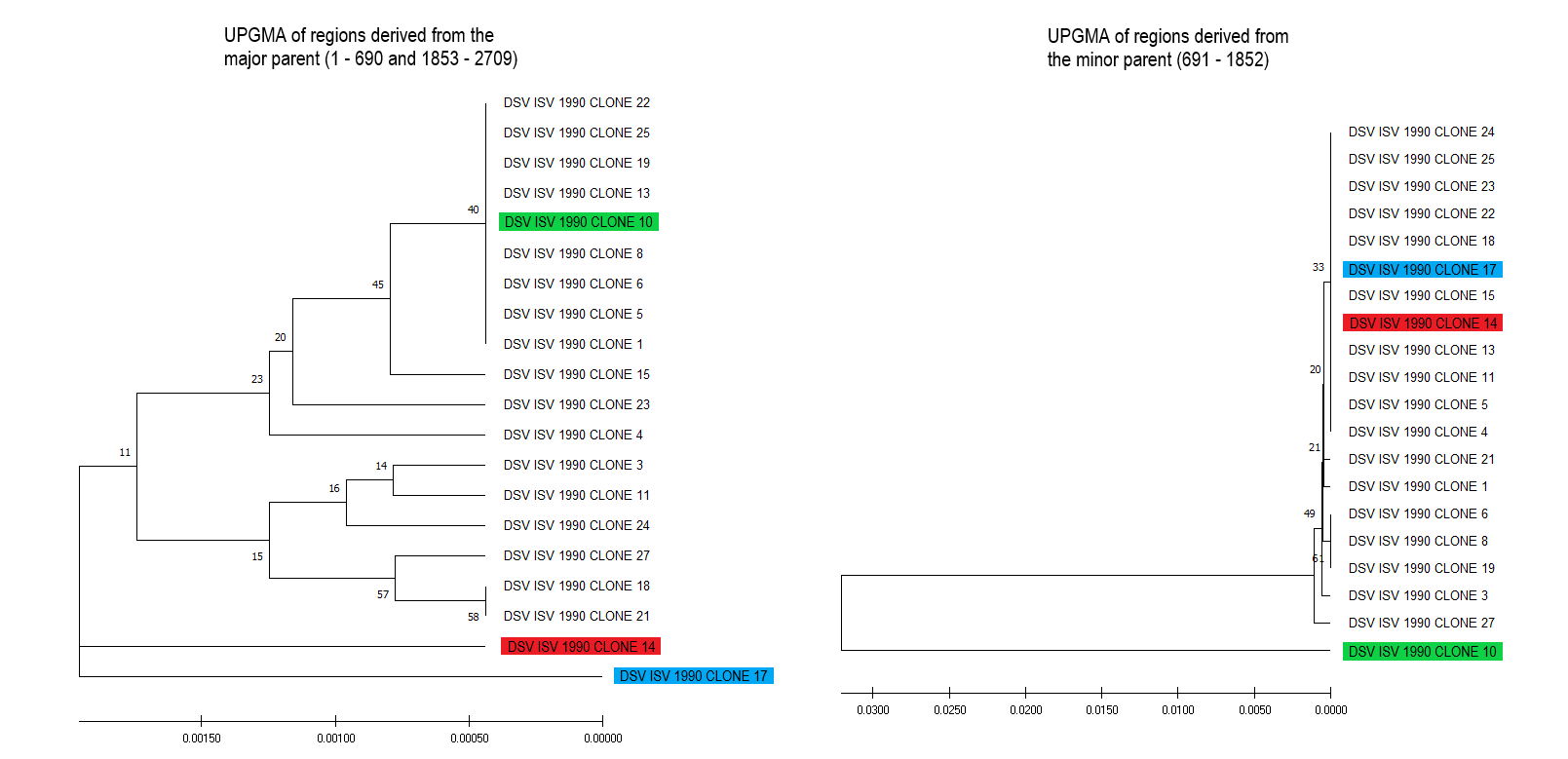


**Figure S7.1.** Phylogeny constructions in UPGMA of the ISV 1990 mutant spectrum. Using RDP4 software, clone 10 was determined to be a recombinant sequence (green box). The major parental sequence was clone 14 (red box) and the minor parental sequence was clone 17 (blue box). To validate the recombination detection results, two UPGMA trees based on Kimura 2-parameter were constructed using the MEGA-X program, using: one with the regions derived from the major parent of the recombinant sequence (1-690 and 1853-2709) and one with the regions derived from the minor parent of the recombinant sequence (691-1852).


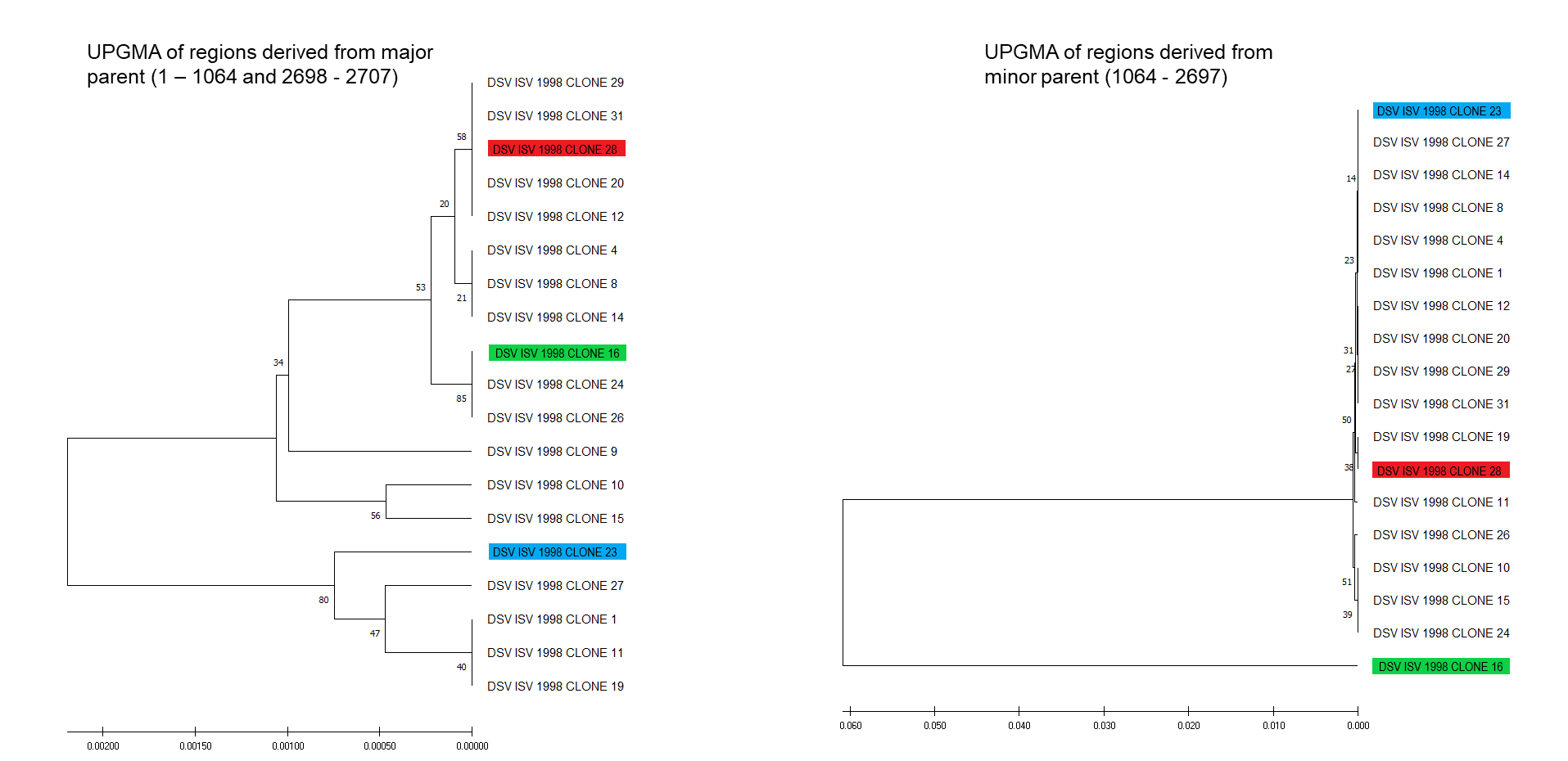


**Figure S7.2.** Phylogeny constructions in UPGMA of the ISV 1998 mutant spectrum. Using RDP4 software, clone 16 was determined to be a recombinant sequence (green box). The major parental sequence was clone 28 (red box) and the minor parental sequence was clone 23 (blue box). To validate the recombination detection results, two UPGMA trees based on Kimura 2-parameter were constructed using the MEGA-X program, using: one with the regions derived from the major parent of the recombinant sequence (1-1064 and 2698-2707) and one with the regions derived from the minor parent of the recombinant sequence (1065-2696).
