# Supplementary material for: Twenty years of evolution and diversification of digitaria streak virus in Digitaria setigera
Source: Virus Evol. 2021 Oct 13;7(2):veab083. doi: 10.1093/ve/veab083 (PMC8516820; doi:10.1093/ve/veab083)
Supplement: veab083_Supp [file veab083_supp.zip › Supplementary Table S8_Ortega del Campo_VE.docx]

**Supplementary Table S8.** Table containing the GenBank Accession Numbers of DSV sequences of this study.

|  | **Accession Number** | **Sequence** |
| --- | --- | --- |
| **Consensus sequences** | MW700085 | DSV ISV 1990 |
|  | MW700086 | DSV ISV 1998 |
|  | MW700087 | DSV ISV 1999-01 |
|  | MW700088 | DSV ISV 1999-07 |
|  | MW700089 | DSV ISV 1999-12 |
|  | MW700090 | DSV ISV 2001 |
|  | MW700091 | DSV ISV 2008-B2 |
|  | MW700092 | DSV ISV 2008-B1 |
|  | MW700093 | DSV ISV 2008-B1-FTA |
|  | MW700094 | DSV ISV 2008-C |
|  | MW700095 | DSV ISV 2008-A |
|  | MW700096 | DSV ISV 2008-B |
|  | MW700097 | DSV IVV 2008 |
|  | MW700098 | DSV IVV 2008-FTA |
|  | MW700099 | DSV CIRAD 2010 |
| **Mutant spectrum ISV 1990** | MW700100 | DSV ISV 1990 CLONE 1 |
|  | MW700101 | DSV ISV 1990 CLONE 3 |
|  | MW700102 | DSV ISV 1990 CLONE 4 |
|  | MW700103 | DSV ISV 1990 CLONE 5 |
|  | MW700104 | DSV ISV 1990 CLONE 6 |
|  | MW700105 | DSV ISV 1990 CLONE 8 |
|  | MW700106 | DSV ISV 1990 CLONE 10 |
|  | MW700107 | DSV ISV 1990 CLONE 11 |
|  | MW700108 | DSV ISV 1990 CLONE 13 |
|  | MW700109 | DSV ISV 1990 CLONE 14 |
|  | MW700110 | DSV ISV 1990 CLONE 15 |
|  | MW700111 | DSV ISV 1990 CLONE 17 |
|  | MW700112 | DSV ISV 1990 CLONE 18 |
|  | MW700113 | DSV ISV 1990 CLONE 19 |
|  | MW700114 | DSV ISV 1990 CLONE 21 |
|  | MW700115 | DSV ISV 1990 CLONE 22 |
|  | MW700116 | DSV ISV 1990 CLONE 23 |
|  | MW700117 | DSV ISV 1990 CLONE 24 |
|  | MW700118 | DSV ISV 1990 CLONE 25 |
|  | MW700119 | DSV ISV 1990 CLONE 27 |
| **Mutant spectrum ISV 1998** | MW700120 | DSV ISV 1998 CLONE 1 |
|  | MW700121 | DSV ISV 1998 CLONE 4 |
|  | MW700122 | DSV ISV 1998 CLONE 8 |
|  | MW700123 | DSV ISV 1998 CLONE 9 |
|  | MW700124 | DSV ISV 1998 CLONE 10 |
|  | MW700125 | DSV ISV 1998 CLONE 11 |
|  | MW700126 | DSV ISV 1998 CLONE 12 |
|  | MW700127 | DSV ISV 1998 CLONE 14 |
|  | MW700128 | DSV ISV 1998 CLONE 15 |
|  | MW700129 | DSV ISV 1998 CLONE 16 |
|  | MW700130 | DSV ISV 1998 CLONE 19 |
|  | MW700131 | DSV ISV 1998 CLONE 20 |
|  | MW700132 | DSV ISV 1998 CLONE 23 |
|  | MW700133 | DSV ISV 1998 CLONE 24 |
|  | MW700134 | DSV ISV 1998 CLONE 26 |
|  | MW700135 | DSV ISV 1998 CLONE 27 |
|  | MW700136 | DSV ISV 1998 CLONE 28 |
|  | MW700137 | DSV ISV 1998 CLONE 29 |
|  | MW700138 | DSV ISV 1998 CLONE 31 |
| **Mutant spectrum ISV 2001** | MW700139 | DSV ISV 2001 CLONE 2 |
|  | MW700140 | DSV ISV 2001 CLONE 3 |
|  | MW700141 | DSV ISV 2001 CLONE 6 |
|  | MW700142 | DSV ISV 2001 CLONE 7 |
|  | MW700143 | DSV ISV 2001 CLONE 9 |
|  | MW700144 | DSV ISV 2001 CLONE 11 |
|  | MW700145 | DSV ISV 2001 CLONE 12 |
|  | MW700146 | DSV ISV 2001 CLONE 14 |
|  | MW700147 | DSV ISV 2001 CLONE 15 |
|  | MW700148 | DSV ISV 2001 CLONE 16 |
|  | MW700149 | DSV ISV 2001 CLONE 18 |
|  | MW700150 | DSV ISV 2001 CLONE 19 |
|  | MW700151 | DSV ISV 2001 CLONE 21 |
|  | MW700152 | DSV ISV 2001 CLONE 22 |
|  | MW700153 | DSV ISV 2001 CLONE 23 |
|  | MW700154 | DSV ISV 2001 CLONE 24 |
|  | MW700155 | DSV ISV 2001 CLONE 25 |
|  | MW700156 | DSV ISV 2001 CLONE 26 |
|  | MW700157 | DSV ISV 2001 CLONE 27 |
|  | MW700158 | DSV ISV 2001 CLONE 28 |
|  | MW700159 | DSV ISV 2001 CLONE 29 |
|  | MW700160 | DSV ISV 2001 CLONE 30 |
|  | MW700161 | DSV ISV 2001 CLONE 31 |
| **Mutant spectrum ISV 2008-B2** | MW700162 | DSV ISV 2008-B2 CLONE 1 |
|  | MW700163 | DSV ISV 2008-B2 CLONE 2 |
|  | MW700164 | DSV ISV 2008-B2 CLONE 3 |
|  | MW700165 | DSV ISV 2008-B2 CLONE 4 |
|  | MW700166 | DSV ISV 2008-B2 CLONE 6 |
|  | MW700167 | DSV ISV 2008-B2 CLONE 7 |
|  | MW700168 | DSV ISV 2008-B2 CLONE 9 |
|  | MW700169 | DSV ISV 2008-B2 CLONE 10 |
|  | MW700170 | DSV ISV 2008-B2 CLONE 11 |
|  | MW700171 | DSV ISV 2008-B2 CLONE 12 |
|  | MW700172 | DSV ISV 2008-B2 CLONE 13 |
|  | MW700173 | DSV ISV 2008-B2 CLONE 14 |
|  | MW700174 | DSV ISV 2008-B2 CLONE 15 |
|  | MW700175 | DSV ISV 2008-B2 CLONE 16 |
|  | MW700176 | DSV ISV 2008-B2 CLONE 17 |
|  | MW700177 | DSV ISV 2008-B2 CLONE 18 |
|  | MW700178 | DSV ISV 2008-B2 CLONE 20 |
|  | MW700179 | DSV ISV 2008-B2 CLONE 21 |
|  | MW700180 | DSV ISV 2008-B2 CLONE 23 |
|  | MW700181 | DSV ISV 2008-B2 CLONE 24 |
| **Mutant spectrum ISV 2008-B1** | MW700182 | DSV ISV 2008-B1 CLONE 1 |
|  | MW700183 | DSV ISV 2008-B1 CLONE 5 |
|  | MW700184 | DSV ISV 2008-B1 CLONE 7 |
|  | MW700185 | DSV ISV 2008-B1 CLONE 8 |
|  | MW700186 | DSV ISV 2008-B1 CLONE 12 |
|  | MW700187 | DSV ISV 2008-B1 CLONE 13 |
|  | MW700188 | DSV ISV 2008-B1 CLONE 14 |
|  | MW700189 | DSV ISV 2008-B1 CLONE 17 |
|  | MW700190 | DSV ISV 2008-B1 CLONE 35 |
|  | MW700191 | DSV ISV 2008-B1 CLONE 36 |
|  | MW700192 | DSV ISV 2008-B1 CLONE 37 |
|  | MW700193 | DSV ISV 2008-B1 CLONE 38 |
|  | MW700194 | DSV ISV 2008-B1 CLONE 39 |
|  | MW700195 | DSV ISV 2008-B1 CLONE 40 |
|  | MW700196 | DSV ISV 2008-B1 CLONE 41 |
|  | MW700197 | DSV ISV 2008-B1 CLONE 42 |
|  | MW700198 | DSV ISV 2008-B1 CLONE 43 |
|  | MW700199 | DSV ISV 2008-B1 CLONE 45 |
|  | MW700200 | DSV ISV 2008-B1 CLONE 46 |
|  | MW700201 | DSV ISV 2008-B1 CLONE 48 |
|  | MW700202 | DSV ISV 2008-B1 CLONE 49 |
|  | MW700203 | DSV ISV 2008-B1 CLONE 50 |
| **Mutant spectrum ISV 2008-B1-FTA** | MW700204 | DSV ISV 2008-B1-FTA CLONE 1 |
|  | MW700205 | DSV ISV 2008-B1-FTA CLONE 2 |
|  | MW700206 | DSV ISV 2008-B1-FTA CLONE 3 |
|  | MW700207 | DSV ISV 2008-B1-FTA CLONE 4 |
|  | MW700208 | DSV ISV 2008-B1-FTA CLONE 6 |
|  | MW700209 | DSV ISV 2008-B1-FTA CLONE 7 |
|  | MW700210 | DSV ISV 2008-B1-FTA CLONE 8 |
|  | MW700211 | DSV ISV 2008-B1-FTA CLONE 9 |
|  | MW700212 | DSV ISV 2008-B1-FTA CLONE 10 |
|  | MW700213 | DSV ISV 2008-B1-FTA CLONE 11 |
|  | MW700214 | DSV ISV 2008-B1-FTA CLONE 12 |
|  | MW700215 | DSV ISV 2008-B1-FTA CLONE 13 |
|  | MW700216 | DSV ISV 2008-B1-FTA CLONE 14 |
|  | MW700217 | DSV ISV 2008-B1-FTA CLONE 15 |
|  | MW700218 | DSV ISV 2008-B1-FTA CLONE 16 |
|  | MW700219 | DSV ISV 2008-B1-FTA CLONE 17 |
|  | MW700220 | DSV ISV 2008-B1-FTA CLONE 18 |
|  | MW700221 | DSV ISV 2008-B1-FTA CLONE 19 |
|  | MW700222 | DSV ISV 2008-B1-FTA CLONE 20 |
| **Mutant spectrum IVV 2008** | MW700223 | DSV IVV 2008 CLONE 2 |
|  | MW700224 | DSV IVV 2008 CLONE 3 |
|  | MW700225 | DSV IVV 2008 CLONE 4 |
|  | MW700226 | DSV IVV 2008 CLONE 5 |
|  | MW700227 | DSV IVV 2008 CLONE 6 |
|  | MW700228 | DSV IVV 2008 CLONE 7 |
|  | MW700229 | DSV IVV 2008 CLONE 10 |
|  | MW700230 | DSV IVV 2008 CLONE 11 |
|  | MW700231 | DSV IVV 2008 CLONE 13 |
|  | MW700232 | DSV IVV 2008 CLONE 14 |
|  | MW700233 | DSV IVV 2008 CLONE 15 |
|  | MW700234 | DSV IVV 2008 CLONE 16 |
|  | MW700235 | DSV IVV 2008 CLONE 17 |
|  | MW700236 | DSV IVV 2008 CLONE 18 |
|  | MW700237 | DSV IVV 2008 CLONE 19 |
|  | MW700238 | DSV IVV 2008 CLONE 20 |
|  | MW700239 | DSV IVV 2008 CLONE 21 |
|  | MW700240 | DSV IVV 2008 CLONE 22 |
|  | MW700241 | DSV IVV 2008 CLONE 24 |
|  | MW700242 | DSV IVV 2008 CLONE 25 |
| **Mutant spectrum CIRAD 2010** | MW700243 | DSV CIRAD 2010 CLONE 2 |
|  | MW700244 | DSV CIRAD 2010 CLONE 3 |
|  | MW700245 | DSV CIRAD 2010 CLONE 4 |
|  | MW700246 | DSV CIRAD 2010 CLONE 8 |
|  | MW700247 | DSV CIRAD 2010 CLONE 9 |
|  | MW700248 | DSV CIRAD 2010 CLONE 10 |
|  | MW700249 | DSV CIRAD 2010 CLONE 11 |
|  | MW700250 | DSV CIRAD 2010 CLONE 12 |
|  | MW700251 | DSV CIRAD 2010 CLONE 13 |
|  | MW700252 | DSV CIRAD 2010 CLONE 14 |
|  | MW700253 | DSV CIRAD 2010 CLONE 15 |
|  | MW700254 | DSV CIRAD 2010 CLONE 16 |
|  | MW700255 | DSV CIRAD 2010 CLONE 19 |
|  | MW700256 | DSV CIRAD 2010 CLONE 20 |
|  | MW700257 | DSV CIRAD 2010 CLONE 21 |
|  | MW700258 | DSV CIRAD 2010 CLONE 22 |
|  | MW700259 | DSV CIRAD 2010 CLONE 24 |
|  | MW700260 | DSV CIRAD 2010 CLONE 25 |
|  | MW700261 | DSV CIRAD 2010 CLONE 26 |
|  | MW700262 | DSV CIRAD 2010 CLONE 28 |
|  | MW700263 | DSV CIRAD 2010 CLONE 29 |
|  | MW700264 | DSV CIRAD 2010 CLONE 30 |
